# Amino acid substitutions at rheostat positions in the Na^+^/taurocholate cotransporting polypeptide substrate channel have pleiotropic effects

**DOI:** 10.1002/pro.70313

**Published:** 2025-10-14

**Authors:** Liskin Swint‐Kruse, Melissa J. Ruggiero, Bruno Hagenbuch

**Affiliations:** ^1^ Department of Biochemistry and Molecular Biology The University of Kansas Medical Center Kansas City Kansas USA; ^2^ Department of Pharmacology, Toxicology, and Therapeutics The University of Kansas Medical Center Kansas City Kansas USA; ^3^ Present address: Attentive Science LLC Stilwell Kansas USA

**Keywords:** Na^+^/taurocholate cotransporting polypeptide, site‐directed mutagenesis, transporter function and expression

## Abstract

In the human Na^+^/taurocholate cotransporting polypeptide (NTCP), several non‐synonymous SNPs are known to have positive or negative medical consequences, and hundreds more are “variants of unknown significance.” One reason outcomes remain unknown is that computational predictions are not yet reliable. Such predictions are especially problematic for “rheostat” positions, where amino acid substitutions have widely varied outcomes. We previously predicted that NTCP contains multiple rheostat positions for substrate transport. To test this, we selected two positions that—by classical assumptions—would *not* be rheostat positions. Position G102 is highly conserved and buried deep in the substrate channel; as such, most substitutions are expected to be catastrophic. In contrast, position Y146 is non‐conserved with a solvent‐exposed side chain near the channel opening; thus, most substitutions are expected to be well‐tolerated. At each position, we made all 19 substitutions and assessed the effects on substrate transport and cell surface expression. Position G102 tolerated more substitutions than expected and acted as a rheostat position for both parameters; nevertheless, correlations with side chain properties showed that glycine best met the simultaneous and different requirements of surface expression and substrate transport. While most substitutions at position Y146 were neutral, two substitutions unexpectedly abolished cellular expression. Along with previously studied rheostat positions 267 and 271, results suggest that the substrate channel of NTCP is lined with rheostat positions that contribute to multiple NTCP characteristics with pleiotropic effects. Thus, integrating contributions of rheostat positions to function and/or stability computations should improve prediction of NTCP substitution outcomes.

## 1 INTRODUCTION

One of the proteins that maintains enterohepatic circulation of bile acids is the Na^+^/taurocholate cotransporting polypeptide (NTCP; *SLC10A1*) (Claro da Silva et al., 2013; Hagenbuch & Meier, 1994; Slijepcevic et al., 2015), which is expressed at the basolateral membrane of human hepatocytes (Kullak‐Ublick et al., 1997). In the *SLC10A1* gene, several single nucleotide polymorphisms (SNPs) are known to lead to missense mutations with medical consequences. For example, p.A64T, found in 1% of Korean subjects, leads to decreased taurocholate and rosuvastatin uptake but does not alter protein expression (Pan et al., 2011). The p.S267F polymorphism found in ~7% of Asian subjects (Ho et al., 2004) elevates plasma bile acids; interestingly, p.S267F reduces taurocholate transport but increases the uptake of estrone‐3‐sulfate and rosuvastatin (Pan et al., 2011; Ruggiero et al., 2021). Furthermore, this variant is clinically associated with a decreased risk for hepatitis virus infection, cirrhosis, and hepatocellular carcinoma (An et al., 2018; Lee et al., 2017). In addition to these characterized variants, >580 additional missense SNPs have been reported in the gnomAD database for *SLC10A1*, but few have been experimentally assessed. More are being uncovered, and it is not known which will have positive or negative medical consequences.

**FIGURE 1:**
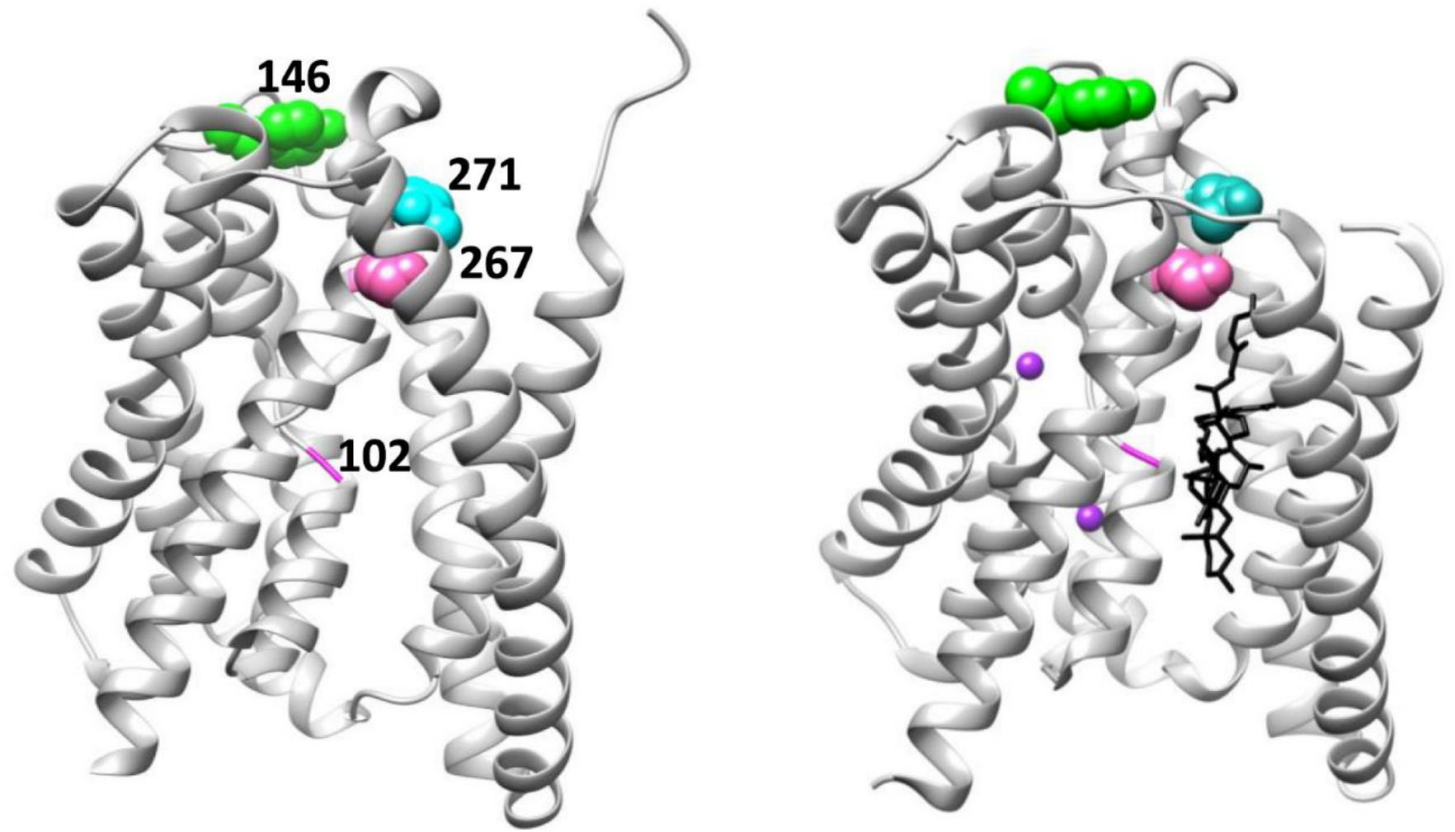
Locations of characterized positions on the inward‐ (left, PDB 7fci; Park et al., 2022) and outward‐open (right, PDB 7zyi; Liu et al., 2022) structures of NTCP. The positions studied are shown in space‐filling models. From top to bottom, they are: Green: Y146 (neutral, ConSurf score 4); cyan: N271 (moderately rheostatic; ConSurf score 5; Ruggiero et al., 2022); pink: S267 (highly rheostatic, substrate specificity altered, ConSurf score 8; Ruggiero et al., 2021); magenta ribbon in the middle: G102 (rheostatic in both transport and surface expression, ConSurf score 9). ConSurf scores interpret a position's sequence entropy in the context of a phylogenetic tree constructed from an MSA (Ashkenazy et al., 2016; Landau et al., 2005; Yariv et al., 2023); scores values range from 1 (least conserved) to 9 (most conserved); positions with intermediate values show conservation in subfamilies but vary across the whole MSA. The outward open NTCP structure on the right also depicts the substrate glycochenodeoxycholic acid in black and two sodium ions with purple spheres. These figures were rendered with UCSF Chimera (Pettersen et al., 2004).

The NTCP structure provides important context to begin to understand the potential medical relevance of missense SNPs. Human NTCP is a 56 kDa glycosylated transporter protein comprising 349 amino acids with nine transmembrane domains flanked by soluble extracellular N‐ and intracellular C‐terminal domains (Liu et al., 2022; Shneider et al., 1997). NTCP‐mediated transport is sodium‐dependent and moves two sodium ions with each bile acid molecule (Anwer & Stieger, 2014). The interaction sites for the two sodium ions and bile acids were recently identified in cryo‐EM structures of human and rat NTCP; the structures of both inward‐ and outward‐open conformations are available (Liu et al., 2022; Park et al., 2022) (Figure 1).

However, the structure alone is insufficient for understanding the outcomes of NTCP variants. For example, the previously characterized p.A65T, p.I223T, p.I279T, p.K314E, and the sets of 19 substitutions at positions 267 and 271 (hereafter referred to as “S267X” and “N271X”) are all capable of measurable transport (Ho et al., 2004; Pan et al., 2011; Ruggiero et al., 2021; Ruggiero et al., 2022). As such, these amino acid changes are *not* likely to cause major structural disruptions, and this suggests that other biophysical features of the protein are altered to modulate transport.

One clue may come from the S267X and N271X variants: At these positions, the sets of substitutions sample a wide range of transport values. As such, these positions meet the criteria of “rheostat” positions (Fenton et al., 2020; Meinhardt et al., 2013; Swint‐Kruse & Fenton, 2024), where different amino acid substitutions can cause gain‐of‐function, neutral, or partial‐ or complete‐loss‐of‐function. Other complex phenotypes, such as altered substrate selectivity, have also been revealed for rheostat positions (Fenton et al., 2020; Ruggiero et al., 2021; Swint‐Kruse & Fenton, 2024). To understand the biophysical basis by which rheostat positions modulate NTCP function, we previously modeled all possible amino acid substitutions at fourteen buried positions on both the inward‐ and outward‐open conformations of NTCP (Figure S1). Results suggested that high structural plasticity allows NTCP to widely tolerate amino acid substitutions without globally unfolding the protein (Ruggiero et al., 2022).

The high plasticity observed in these models also led us to hypothesize that NTCP contains numerous rheostat positions. In turn, this suggests that existing algorithms for predicting SNP effects on NTCP function would perform very poorly, since such algorithms perform poorly for rheostat positions in other proteins (Miller et al., 2017). To further assess the prevalence of NTCP rheostat positions, for this work, we selected two positions that, by classical assumptions, would *not* behave as rheostat positions. First, we searched for a highly conserved and thus “evolutionarily important” position (G102), which we reasoned would be most likely to behave like the classical “toggle” position that does not tolerate any substitutions (Meinhardt et al., 2013; Miller et al., 2017). Second, we searched for a position that was less conserved and solvent exposed on an exoplasmic loop (Y146), hypothesizing that its substitutions would be well tolerated with little to no impact on the function of NTCP and, therefore, be classified as a “neutral” position (Martin et al., 2020; Meinhardt et al., 2013).

Results showed that, although both positions trended toward their expected outcomes, both retained significant rheostatic character for substrate transport. Position 102 also showed rheostatic effects on the amount of surface‐expressed protein. This indicates that substitutions at position 102 have pleiotropic outcomes that differ from the pleiotropic effects on substrate specificity observed for S267X (Ruggiero et al., 2021). Together, these results suggest that either the channel itself or the whole of the NTCP integral membrane domain is enriched with rheostat positions. Thus, successful computational predictions for *SLC10A1* missense SNPs must account for the complex functional outcomes that arise from substituting rheostat positions in NTCP.

## 2 RESULTS AND DISCUSSION

### 2.1 Position selection

When looking for non‐rheostat positions in NTCP, we used a selection strategy based on the following common assumptions: Highly conserved positions are critical to structure/function and cannot tolerate substitutions; highly solvent‐exposed positions can tolerate most substitutions without altering structure/function. For this selection, we utilized the previously published multiple sequence alignment (MSA) that contained homologs from humans through bacteria to calculate sequence entropy and carry out ConSurf analyses (Ruggiero et al., 2021), as well as models of NTCP that were built before the cryo‐EM structures became available (Ruggiero et al., 2022).

These analyses showed that position 102, which has a wild‐type glycine residue, is one of the most evolutionarily conserved positions in NTCP. Such a signature is usually interpreted as the glycine at position 102 being critical for protein function and/or stability; any substitution at position 102 is predicted to be highly detrimental, if not catastrophic. Since this outcome would be expected for most (if not all) of the possible 19 substitutions, this position's overall substitution sensitivity would be expected to exhibit a “toggle” outcome (Landau et al., 2005). Notably, position 102 was chosen for this study based only on its evolutionary information. Nonetheless, the model and cryo‐EM structures revealed that this position is located deep in the substrate channel (Figure 1), which would also be expected to be highly sensitive to amino acid substitutions.

In contrast, tyrosine 146 is much less conserved. In addition, our structural models predicted that position 146 would be in an extracellular, solvent‐exposed loop (Ruggiero et al., 2021; Ruggiero et al., 2022), which was confirmed on the cryo‐EM structures. Such a location is often expected to widely tolerate substitutions with little to no impact on the function or stability. As such, this position would be predicted to be a neutral position (Martin et al., 2020).

### 2.2 Cellular substrate uptake by G102X and Y146X

**FIGURE 2:**
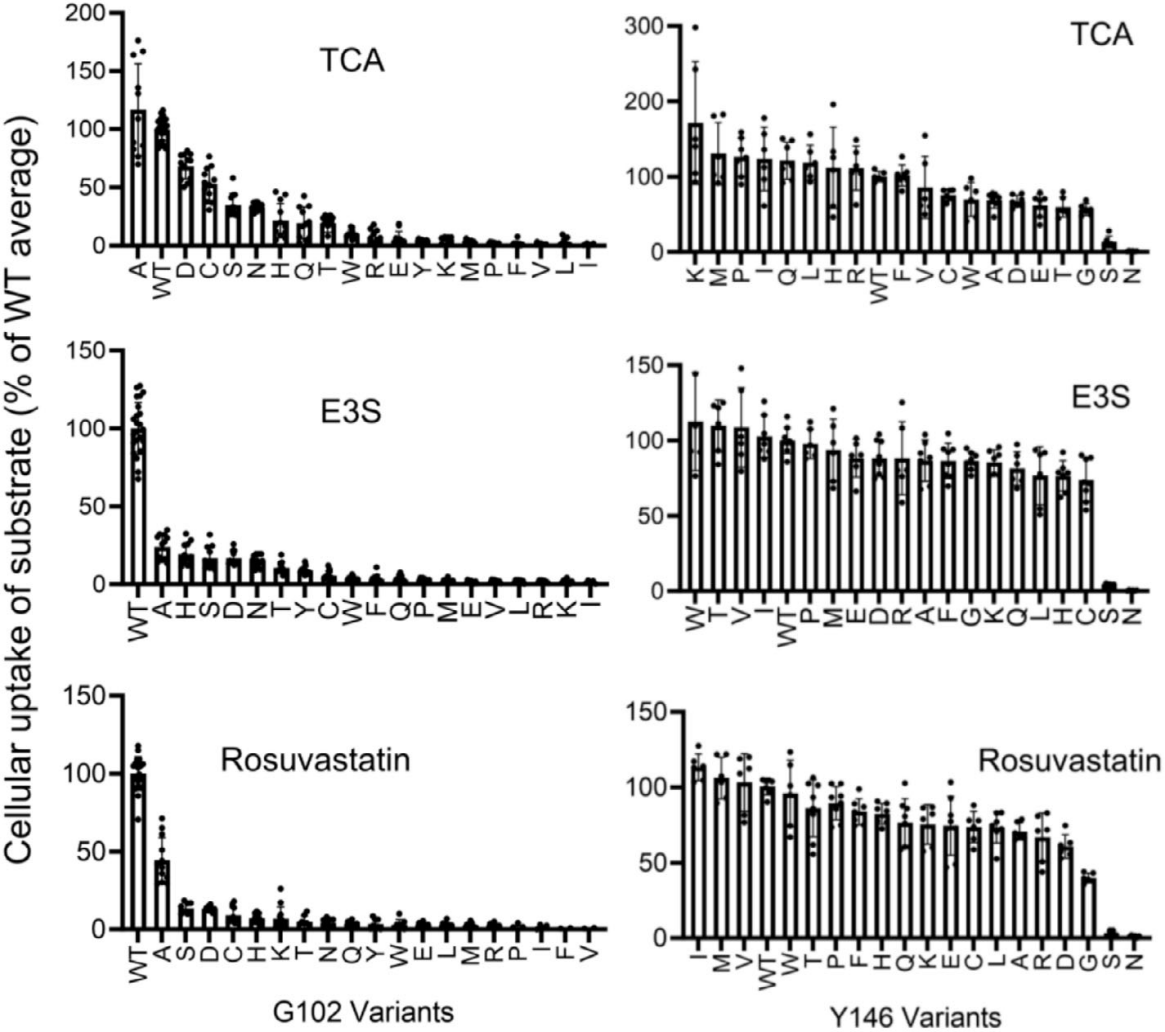
Cellular uptake of substrates by wildtype NTCP, G102X, and Y146X variants. Transport of radiolabeled taurocholate (TCA), estrone‐3‐sulfate (E3S), and rosuvastatin was measured using cells transfected with empty vector, wildtype NTCP (WT), G102X (left), and Y146X (right) variants. Net uptake was calculated by subtracting values from cells transfected with empty vector from the transport by WT and substitution variants. Results for the substitutions are shown in order from greatest to least transport. These cellular uptake values are not normalized for NTCP expression. Individual data points are biological replicates from three individually measured experiments with at least two to three technical replicates each. Mean results are indicated by the bars, and error bars represent the standard deviations. Transport for each substitution is shown as a percent relative to the WT average, which was set to 100%.

We substituted glycine at position 102 and tyrosine at position 146 with all 19 other amino acids and examined changes in cellular uptake for three substrates: taurocholate, estrone‐3‐sulfate, and rosuvastatin (Figure 2). The two sets of substitutions are hereafter referred to as G102X and Y146X. Taurocholate (taurocholic acid) is one of the main bile acids transported by NTCP (Doring et al., 2012). Estrone‐3‐sulfate represents a sulfated steroid hormone substrate (Claro da Silva et al., 2013), while rosuvastatin, a cholesterol‐lowering drug, is commonly used to represent the xenobiotics transported by NTCP (Ho et al., 2006). All results for all substitutions are reported in Table S1.

The set of substitutions at position 102 showed rheostatic effects on taurocholate uptake, while uptake for estrone‐3‐sulfate and rosuvastatin was greatly diminished by most substitutions (Figure 2). Of the substitutions with measurable function, the wildtype glycine was the best for each substrate, except for G102A's cellular uptake of taurocholate. In contrast, most substitutions at Y146 showed few effects on the uptake of any tested substrate (Figure 2), with the exceptions of Y146S and Y146N which lacked the ability to uptake any substrate. Intriguingly, a recently published study confirmed that the Y146A mutation in combination with F274A retained bile acid transport but showed reduced hepatitis B infection (Kunz et al., 2025); we hypothesize that other amino acid substitutions at Y146 will alter viral uptake, perhaps with rheostatic outcomes.

### 2.3 Surface expression of G102X and Y146X

Substitutions that led to diminished cellular substrate uptake could have either impaired transport and/or decreased expression on the cell surface. Changes in surface expression can arise from several sources, including alterations in protein folding or stability, NTCP's insertion into the membrane, post‐translational modifications (like glycosylation), and/or intracellular trafficking from the endoplasmic reticulum to the extracellular surface. Glycosylation, in particular, is necessary for trafficking NTCP to the cell membrane and disrupting NTCP glycosylation can lead to endocytosis and lysosomal degradation (Appelman et al., 2017). Decreased NTCP glycosylation has also been reported in patients with non‐alcoholic steatohepatitis (Clarke et al., 2017) and impairs NTCP's receptor activity for the Hepatitis B/D virus at the cell surface (Appelman et al., 2017).

**FIGURE 3:**
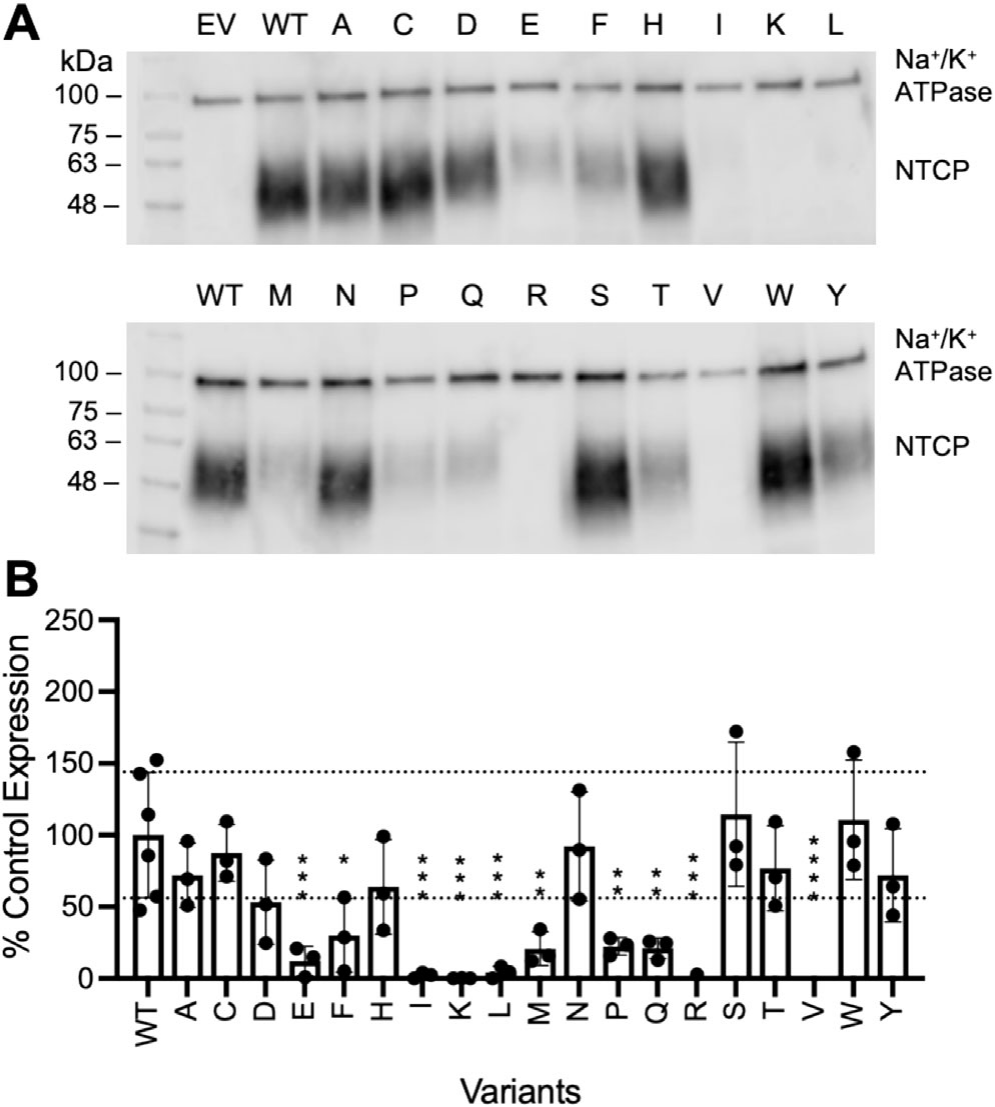
Surface expression and quantification of wildtype NTCP and G102 variants. (a) A representative western blot of the surface expression of wildtype (WT) NTCP and G102 variants; “EV” is empty vector. Blots were probed simultaneously with Na^+^/K^+^‐ATPase (100 kDa) as a loading control and Tetra‐His antibodies, which detect His‐tagged proteins. (b) Quantification of wildtype (WT) and G102 variant surface expression. Three independent surface expression experiments were quantified using Image Studio Lite, and individual data points are shown with the bar graphs representing the mean ± SD. Horizontal lines are shown at the upper and lower limits of the wildtype standard deviation. *p < 0.05, **p < 0.005, ***p < 0.001 compared to wildtype NTCP.

**FIGURE 4:**
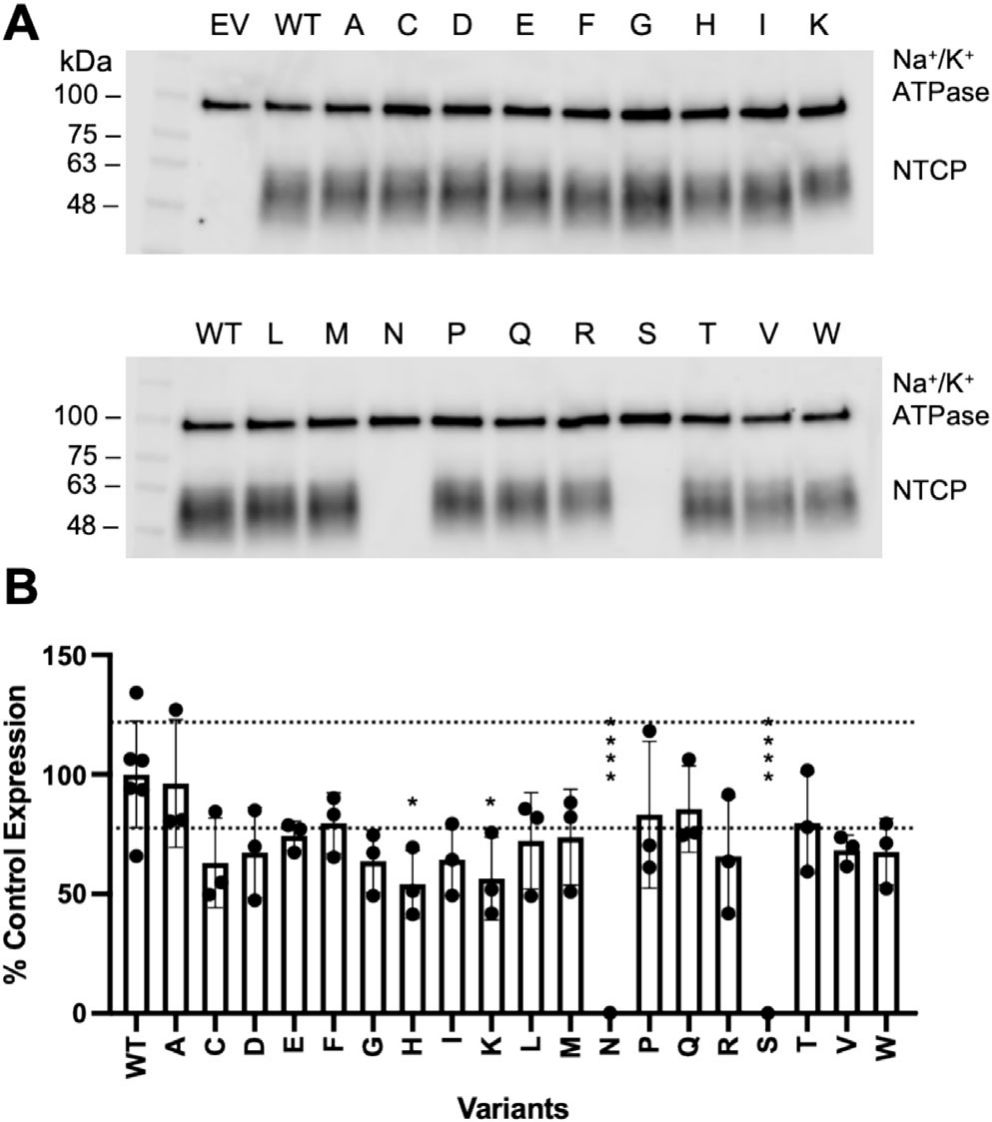
Surface expression and quantification of wildtype NTCP and Y146 variants. Experiments like those in Figure 3 were completed with the Y146 variants. In short, (a) surface expression of wildtype (WT) NTCP and Y146 variants on a representative western blot; “EV” is empty vector. Blots were probed simultaneously with Na^+^/K^+^‐ATPase (100 kDa) as a loading control and Tetra‐His antibodies, which detect His‐tagged proteins. (b) Quantification of wildtype (WT) and Y146 variant surface expression. Three independent surface expression experiments were quantified using Image Studio Lite, and individual data points are shown with the bar graphs representing the mean ± SD. Horizontal lines are shown at the upper and lower limits of the wildtype standard deviation. *p < 0.05, ***p < 0.001 compared to wildtype NTCP.

To determine expression levels of the G102X and Y146X variants, we separately measured the amounts expressed on the cell surface as well as the amounts and glycosylation states of the total protein expressed in the cell. Figures 3 and 4 show the result of the surface biotinylation assays, and detailed values are reported in Table S1. For position 102, expression of the isoleucine, leucine, lysine, arginine, and valine substitutions was not detected (Figure 3), which explains why substrate uptake was not detected (Figure 2). Several other substitutions at position 102 showed varied amounts of surface expression, which differs from prior results for amino acid substitutions at positions 267 and 271, which had few effects on surface expression (Ruggiero et al., 2021; Ruggiero et al., 2022). Quantification of the Y146 substitutions revealed that substitutions with serine or asparagine were not expressed at the plasma membrane (Figure 4), which explains the lack of substrate uptake for these two residues (Figure 2).

Western blots of the total protein fractions (Figures S2 and S3) demonstrate that G102X and Y146X substitutions showed different levels of glycosylation. NTCP is N‐glycosylated at residues N5 and N11 and runs as a broad band around 55 kDa, which is reduced to about 35 kDa upon treatment with the deglycosylating enzyme PNGase (Appelman et al., 2017). Only the glycosylated form is seen at the plasma membrane, which was confirmed for both sets of substitutions in the current study. Several G102X variants (G102E, G102F, G102I, G102K, G102L, G102M, G102Q, G102R, and G102V) with diminished surface expression exhibited various states of glycosylation and degradation, which indicates that trafficking to the plasma membrane was affected. Of the two Y146 variants not detected on the cell surface, Y146S was made but not glycosylated, and Y146N was not detectable at all. This indicates that the non‐glycosylated Y146S is not properly trafficked to the membrane, while Y146N is likely degraded during translation or shortly thereafter. Since positions 102 and 146 are located far from the glycosylation sites on the extracellular N‐terminal of NTCP, amino acid substitutions that affect glycosylation likely have long‐range effects.

### 2.4 Corrected substrate transport for G102X and Y146X

**FIGURE 5:**
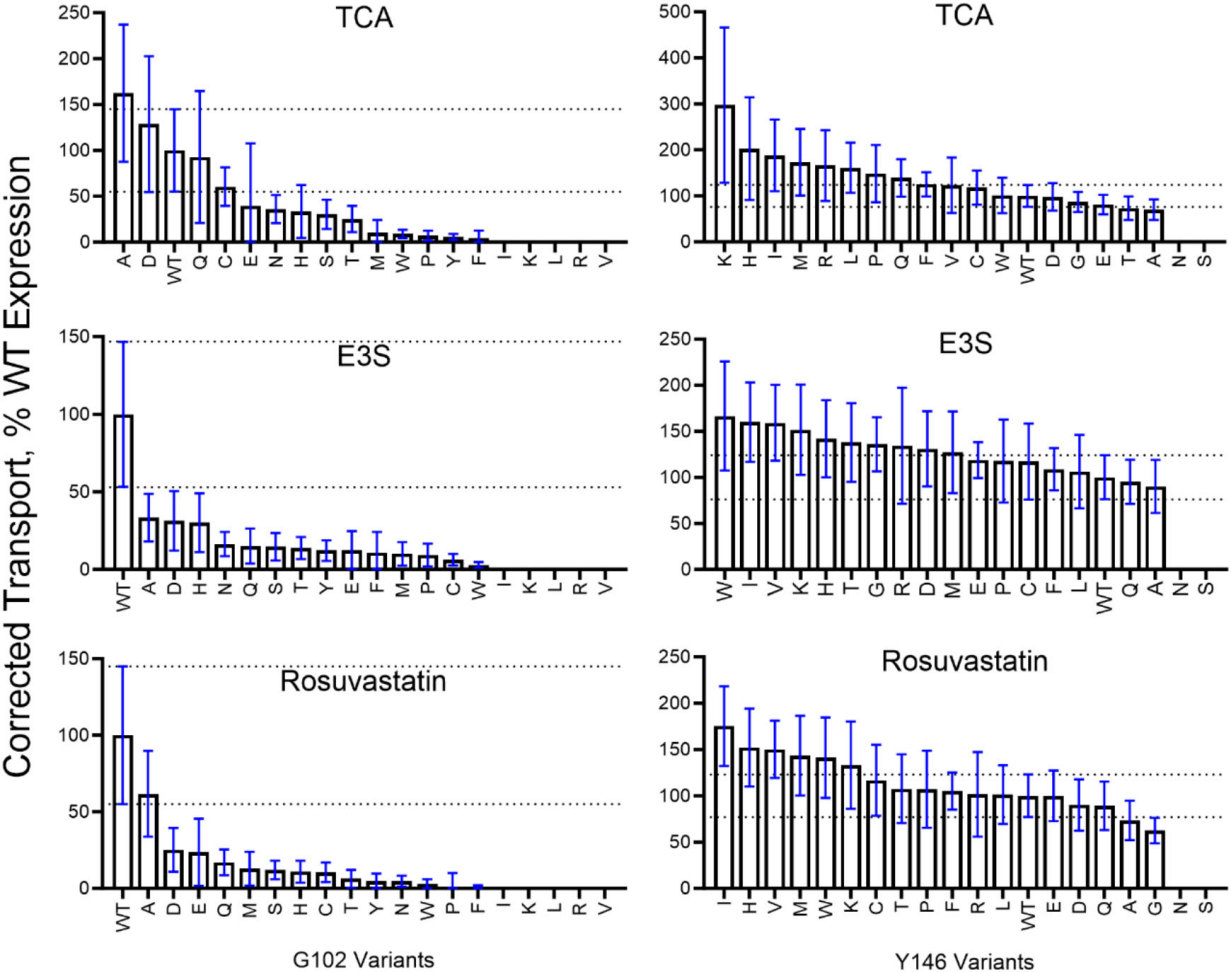
Corrected substrate transport for G102X (left) and Y146X (right) variants. The average values for cellular uptake results (Figure 2) were corrected for the measured surface expression for each variant (Figures 3 and 4). Corrected transport values are indicated by bars, and error bars represent the standard deviations propagated from the standard deviations of the uptake and expression measurements; propagation was carried out in Table S1 and the cells retain the propagation equations. The horizontal dashed lines denote the upper and lower limits for the standard deviation of the WT value. Variants G102I, K, L, R, and V, and Y146N and S were excluded from the calculations because their expression was not detected on the cell surface.

Finally, we used the surface expression (Figures 3 and 4) and cellular uptake (Figure 2) data to isolate effects on transport function that arise from substitutions at positions 102 and 146 (Figure 5). For position 102, several variants with lower expression showed reasonable transport activity when abundance was accounted for (e.g., G102D), whereas others showed altered transport. For position 146, little change was observed after normalization (Figure 4b; Table S1), which was expected since only minor variations were observed in surface expression. In summary, many substitutions at G102 affected transport and/or expression, whereas most Y146 substitutions had few effects on either parameter.

### 2.5 Further analyses of G102X

Since G102X variants with altered surface expression may be due to overall stability, we next considered whether substitutions at this position showed the stability/function “tradeoff” that has been observed in some enzymes (Hou et al., 2023; Khersonsky et al., 2010; Klesmith et al., 2017; Xie et al., 2022; Xie & Warshel, 2022); a common interpretation is that enhanced stability makes the enzyme too rigid to carry out its catalysis. However, no correlations were observed for NTPC position 102 (Figure S4). This suggests that, for individual NTCP proteins, substitution effects on surface expression and transport are independent of each other and that their separate modulation is controlled by changes in different biophysical parameters.

Next, we compared changes in G102X transport and expression to various amino acid properties (Figure S5). Properties evaluated included: hydrophobicity (Wimley et al., 1996), amino acid size was determined from solvent‐exposed surface area (Bendell et al., 2014), and position‐specific helical propensities—which account for the different hydrogen bond patterns that occur for positions in the middle of the helix versus those at its termini (such as the “N‐cap” and “C‐cap” positions) (Kumar & Bansal, 1998).

Position 102 is buried in the middle of NTCP, near the center of the substrate channel and on a small break between two helices (Figure 1). This places G102 at the N‐terminal side of the second helix, which is a potential “N‐cap” position. We hypothesized that amino acid substitutions—which remove the helix‐breaking glycine—could lead to fusion of these helices into one longer helix that would hinder NTCP's stability or transport that is conveyed by having two shorter helices with a short flexible linker. Indeed, comparisons of helical propensities to surface expression showed a Spearman correlation >0.5 for several position‐specific metrics; two of the strongest correlations are shown in Figure S5.

Surface expression did not show a meaningful correlation with the hydrophobicity of the substituted side chain (not shown); however, the substitutions that lack detectable surface expression (V, K, I, L, R)—which are not included on the plot—all have long aliphatic side chains; this suggests that the structure cannot accommodate this feature at this position. This is also consistent with trends observed for substrate transport, which showed a meaningful correlation with amino acid size and hydrophobicity (Figure S5). Large side chains diminished transport, which suggests that large side chains may also block the channel and/or inhibit the inward‐to‐outward‐open transition. Another possibility is that adding aliphatic atoms to this region of NTCP interferes with Na^+^ binding at its nearby site, which is required for transport.

The separate correlations between surface expression and transport support the hypothesis that surface expression and transport have different biophysical bases. These results also suggest the intriguing reason why glycine is a highly conserved amino acid at this position: If the protein must simultaneously satisfy multiple biophysical criteria, neither parameter could have a perfect correlation. Glycine must be the amino acid that best satisfies all the requirements. This also illustrates how a single substitution's pleiotropic effects can emerge from each amino acid's unique—and context dependent—chemical and dynamic characteristics. Even alanine, which is often considered to be the amino acid most similar to glycine, has an aliphatic side chain that makes it both more apolar and larger and restricts the polypeptide backbone's degrees of freedom. The latter could have big effects on the conformational transition between NTCP's inward‐ and outward‐open conformations. Finally, changing even a few atoms can have big effects on the functionally important thermal fluctuations that occur within individual conformations (e.g., Campitelli et al., 2021).

These results for G102 also contrast with the two previously studied NTCP positions for which substitutions altered transport: positions 271 (Ruggiero et al., 2022) and 267 (Ruggiero et al., 2021). We re‐analyzed these data for potential correlations with the properties listed above. The set of substitutions at position 271 only showed a moderate correlation with two of the helical propensity scales (Spearman R = 0.68; Figure S6). For the set of substitutions at position 267, taurocholate transport did not even correlate with the transport of the other two substrates (Ruggiero et al., 2021), which means that any side chain correlation that exists for one substrate would be scrambled for a second substrate—de facto excluding correlations with side chain properties. Nonetheless, we compared the rosuvastatin data with amino acid properties. As expected, no correlations were observed (not shown). The *lack* of correlation for positions 271 and 267 is consistent with observations for rheostat positions in other proteins (Meinhardt et al., 2013; Sreenivasan et al., 2024; Swint‐Kruse & Fenton, 2024). One possibility is that substitutions at these NTCP rheostat positions modulate function by altering dynamics, as we have observed in two other proteins (Campitelli et al., 2021; Campitelli et al., 2024; Kariyawasam et al., 2025).

**FIGURE 6:**
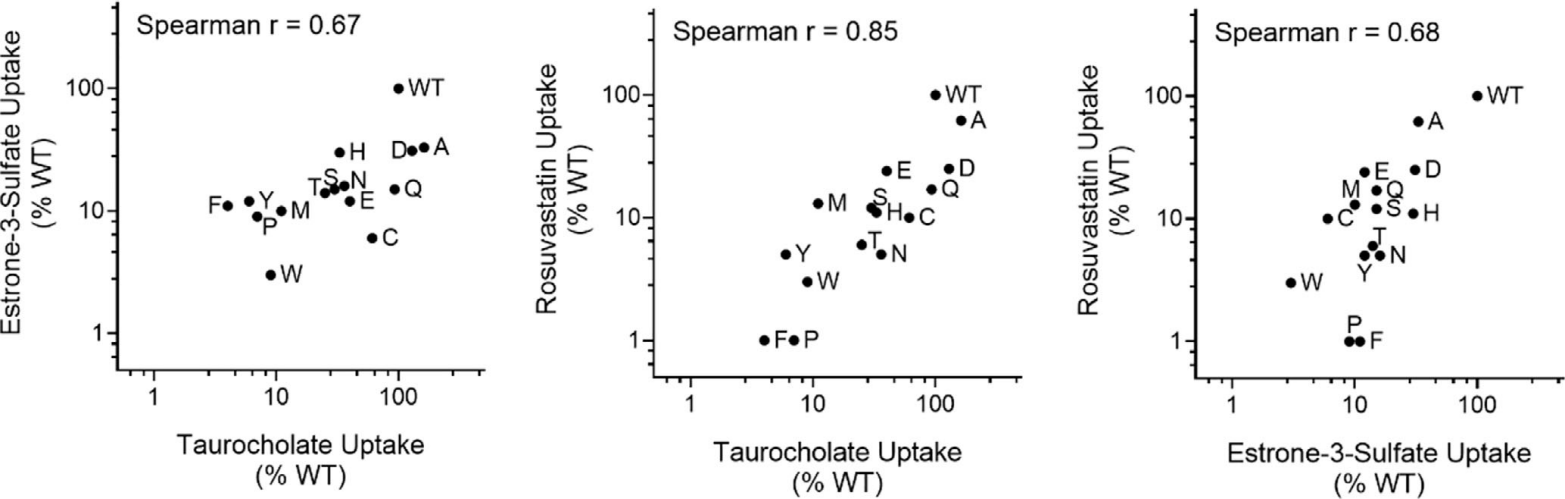
Substitutions at position G102 do not substantially alter substrate specificity. The surface‐corrected transport values (Figure 5) are shown as pairwise plots with Spearman correlation analyses. Plots are shown here on log scale; correlations were performed in Graphpad Prism on the untransformed data.

Finally, we compared the rank orders of the G102X amino acid side chains for the three substrates to assess whether substrate specificity is affected by these changes (Figure 6). If the rank order is similar for alternative substrates, specificity is unchanged. Conversely, if the rank order is altered, substrate specificity is changed (Tungtur et al., 2019). Position 102 showed little change in substrate specificity, especially compared to the changes previously observed for position 267 (Ruggiero et al., 2021).

Another use of the substrate‐versus‐substrate comparison is to assess the reliability of the values with intermediate transport or expression. The corrected transport of the three different substrates shows very similar rank orders (Figure 6), which suggests that the intermediate values are more reliably measured than the propagated error bars of Figure 5 would suggest. The rank order for substitutions at position 271 was also strongly correlated (Ruggiero et al., 2022). Assuming that the reproducibility and reliability for substitutions with intermediate outcomes are similar for our previous and current studies, this gives confidence in the reliability of measured intermediate values for the whole dataset, thereby enabling the RheoScale analyses presented in the next section.

### 2.6 RheoScale calculations and outcomes

To objectively compare the overall substitution sensitivities of NTCP positions 102 and 146 with those of the previously characterized positions 267 (Ruggiero et al., 2021) and 271 (Ruggiero et al., 2022), we quantified each position's overall substitution sensitivity using histogram‐based analyses (Hodges et al., 2018). We previously made subjective assessments for positions 267 and 271; however, with the current data for positions 102 and 146, we now have sufficient information to evaluate them more objectively. In these analyses, if the majority of a position's substitutions are statistically equivalent to wildtype, the position is classified as “neutral” (Martin et al., 2020). In contrast, if a majority of a position's substitutions lack detectable activity or protein expression, then the position is classified as a toggle position (Wu et al., 2019). Finally, if a position's set of substitutions sample at least half of the possible functional range, i.e., at least 50% of the histogram bins are populated by at least one substitution, then the position is considered a rheostat (score of 0.5 or greater) (Hodges et al., 2018). Positions where most substitutions are *not* neutral, but their values sample less than half of the accessible range, have been defined as “moderate” rheostat positions (Swint‐Kruse et al., 2021).

**FIGURE 7:**
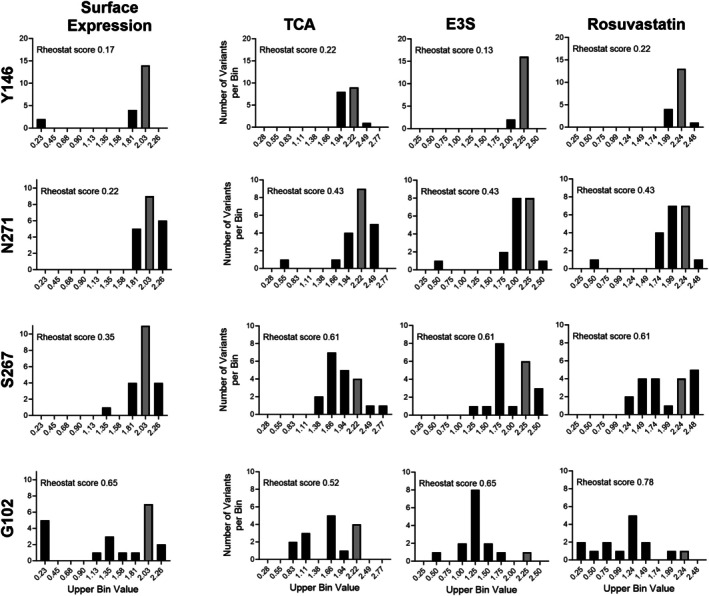
Histograms for WT and 19 substitutions at NTCP positions Y146, N271, S267, and G102 for the experimentally measured surface protein expression and transport of taurocholate (TCA), estrone‐3‐sulfate (E3S), and rosuvastatin. The positions are ordered to correspond to their locations on the NTCP channel (Figure 1). The uptake data were corrected for changes in surface expression. On each plot, the left‐most bin corresponds to the “dead” value and the value of the WT variant is shown with a gray bar. Rheostat scores from RheoScale analyses are shown on each plot. Data for positions 271 and 267 are adapted from (Ruggiero et al., 2021; Ruggiero et al., 2022), with their surface expression values re‐normalized to match those of positions 102 and 146 (see Methods). Substitutions with no surface expression (less than 1%) were excluded from the uptake calculations (G102 substitutions I, K, L, R, and V; Y146 substitutions N and S) since their “functions” cannot be determined.

The rheostat scores for each position are shown in Figure 7. In agreement with our prior assignment, position 267 is a rheostat position for substrate transport. The rheostat scores for position 271 were just below the empirically determined threshold of 0.5, which classifies it as a moderate rheostat position. For the positions assessed in the current work, neither perfectly matched the expectations derived from applying common assumptions: Surface‐exposed and non‐conserved Y146 is a nearly neutral position, but it nevertheless had two substitutions that abolished its expression on the cell surface; the mechanisms of these effects have yet to be elucidated but may be related to altered glycosylation. On the other hand, the highly conserved and buried G102 tolerated substitutions better than expected.

Indeed, position 102 was classified as a rheostat position for all four parameters (surface expression and transport of three substrates). Even when effects on expression and transport were combined in the cellular uptake assay, multiple substitutions at position 102 maintained intermediate transport levels, and its rheostat score for TCA cellular uptake increased to 1.0 (not shown). This indicates that the NTCP structure must be able to accommodate a variety of amino acid side chains at position 102, consistent with the structural plasticity we previously predicted using computational modeling (Ruggiero et al., 2022).

**FIGURE 8:**
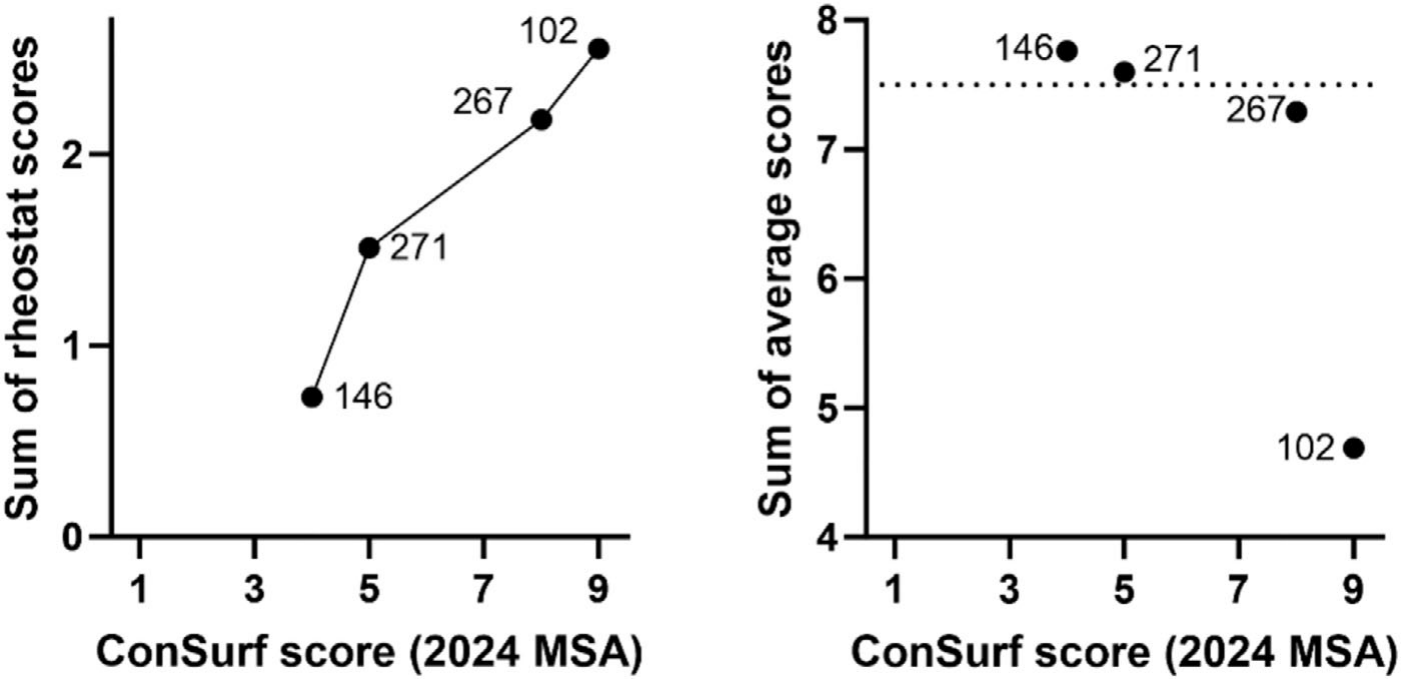
The overall substitution sensitivities of NTCP positions 102, 146, 267, and 271 correlate with their evolutionary ConSurf scores. (Left) Values on the y axis represent the sum of the rheostat scores shown in Figure 7. Values on the x axis correspond to scores from analyses using the updated ConSurf server (see Methods; Yariv et al., 2023); ConSurf values range from 1 (least conserved) to 9 (most conserved). The Pearson correlation coefficient is R = 0.97 with a *p* value of 0.03. The connecting line is to aid visualization of the trend. Despite this correlation, each position contributes to different NTCP characteristics: Substitutions at position 102 alter surface expression and substrate transport; substitutions at position 267 alter substrate transport and substrate specificity; substitutions at position 271 alter substrate transport; substitutions at position 146 have few effects on the assessed characteristics. (Right) Values on the y axis represent the sums of the average outcomes for the four measured parameters (Table S1); the dotted line is to aid visual inspection of the data. The Pearson correlation coefficient is −0.79 with a *p* value of 0.2. Effects at positions 146, 271, and 267 are not as well discriminated by their average outcomes as by their rheostat scores.

Nevertheless, the RheoScale analyses of the four positions did reveal two striking correlations. First, the positions' sensitivity to substitution (Figure 7) showed a clear and expected pattern with their locations in the substrate channel (Figure 1). It will be interesting to determine whether the pattern persists on the cytosolic half of the channel. We also hypothesize that the substrate channel likely contains multiple rheostat positions; this is supported by observations in the macaque NTCP, in which position 158 is a rheostat position for substrate uptake and—intriguingly—a toggle position for entry of the hepatitis B/D virus (Shionoya et al., 2024). Second, the different substitution sensitivities for the four positions in human NTCP also correlated with their phylogenetic scores derived from analyses of the protein family (Figure 8); notably, this distinction was not readily evident from the average substitution outcomes, which are the most common metric used in other site‐saturating substitution studies.

The correlation between these four NTCP positions' substitution sensitivities and their phylogenetic scores is similar to observations for human liver pyruvate kinase and the *Escherichia coli* lactose repressor protein (Swint‐Kruse et al., 2021). These proteins, with data available for more positions, have average ConSurf scores for sets of toggle, rheostat, and neutral positions which show clear trends: the average for the set of toggle positions is the most conserved, the set of rheostat positions has intermediate scores, and the set of neutral positions is least conserved. However, the distribution of ConSurf scores for each position type also significantly overlaps with the distributions of other position types. This overlap could explain why highly conserved NTCP G102, which we initially expected would be a toggle position, is instead a rheostat position. Alternatively, the G102 ConSurf score of 9 could arise because ConSurf scores are internally normalized to the protein studied; ConSurf scores cannot be meaningfully compared across proteins from different families. As such, it is possible that NTCP lacks toggle positions, so that its highest ConSurf scores are assigned to rheostat positions.

Another intriguing observation is that each of the four NTCP positions had substitutions that were more efficient at substrate transport and/or expression than wildtype, which has been observed in many other proteins (Fenton et al., 2020; Hodges et al., 2018; Meinhardt et al., 2013; Sreenivasan et al., 2024; Sreenivasan et al., 2025; Swint‐Kruse & Fenton, 2024; Wu et al., 2019) and may be a common feature of rheostat positions. Perhaps this is not surprising: Evolution does not need to identify the “best” protein, only the ones that are “good enough.” Furthermore, many proteins must simultaneously evolve the “best” function for multiple aspects of function or accommodate the needs of both function and structure (Chi & Liberles, 2016), as appears to be the case here for position 102. Alternatively, disrupting the natural homeostasis of a system by replacing the evolved transporter with a highly efficient one may cause toxicity. Finally, evolution can be influenced by external pressures, such as avoiding the substitutions allowing hepatitis uptake (Shionoya et al., 2024), which provide additional biological “filters.”

## 3 CONCLUSION

The current work supports the hypothesis that rheostat positions are prevalent in NTCP and provides a second example of a rheostat position with pleiotropic effects on function and plasma membrane expression (G102X). We previously hypothesized the existence of such positions (Swint‐Kruse & Fenton, 2024) and recently documented this behavior in the main protease of SARS CoV2 (Sreenivasan et al., 2025). Indeed, the picture is emerging that many rheostat positions have pleiotropic effects. NTCP S267X had pleiotropic effects on function, on the maximal velocity (*V*_max_) and the Michaelis constant (*K*_*m*_) of transport, as well as on substrate specificity, but few effects on expression (Ruggiero et al., 2021). Rheostat positions in human liver pyruvate kinase (Wu et al., 2019) and the *E. coli* lactose repressor protein (Swint‐Kruse & Fenton, 2024) exhibited pleiotropic effects on ligand binding and allosteric regulation. Many of these contributions appear to influence the signals captured in evolutionary information (Figure 8), but because some homologs lack family‐wide characteristics (Page et al., 2022), MSAs themselves are insufficient to fully predict the substitution outcomes of rheostat positions.

Further, for several rheostat positions, their sets of substitutions did *not* show correlations among the parameters assessed (Bantis et al., 2020; Wu et al., 2019); this suggests that various aspects of function/structure are controlled by distinct biophysical processes. For NTCP, the interconversion between the inward‐ and outward‐open conformations is a good candidate for a biophysical process that can be changed by substitutions at rheostat positions. Changes at rheostat positions have been associated with altered dynamics in human aldolase A (Fenton et al., 2022) and the *E. coli* lactose repressor protein (Campitelli et al., 2021; Campitelli et al., 2024; Kariyawasam et al., 2025). For the latter, substitutions at rheostat positions appear to re‐wire the network of thermal fluctuations, without altering the overall protein structure. We can envision a similar scenario for NTCP: substitutions at rheostat positions will re‐wire the network. Substrate ligands will also participate in dynamic fluctuation networks, which would also cause network rewiring in addition to potential changes in the direct bonding network. Combinations of altered substrate and amino acid substitutions could then manifest as altered substrate specificity. Finally, substitution effects on protein stability and/or transport of the protein to the plasma membrane require interacting with glycosylation enzymes and proteins used to chaperone NTCP to the correct location, which might also be sensitive to network re‐wiring by substitutions at rheostat positions.

**FIGURE 9:**
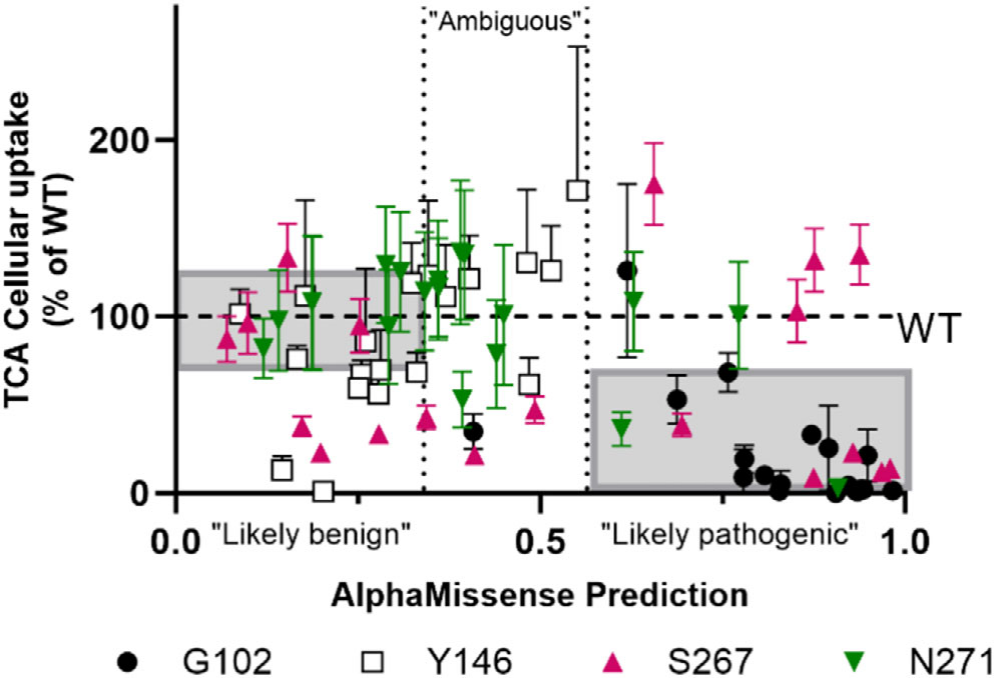
Example of measured versus predicted outcomes for 76 singly generated substitutions of human NTCP. The experimental data plotted were biological data (transport not normalized to surface expression), since AlphaMissense (Cheng et al., 2023) does not discriminate between changes in function and protein abundance. AlphaMissense values for human NTCP were downloaded from the AlphaFold entry Q14973. The gray box in the “benign” region corresponds to the range measured for WT values. The gray box in the “pathogenic” region corresponds to any cellular uptake that is less than WT; we note that some of these values might *not* actually be “pathogenic” in the context of the organism. In the regions labeled “Likely benign” and “Likely pathogenic,” any substitution outside of the gray boxes was mis‐predicted. In the “Ambiguous” region, AlphaMissense does not attempt predictions. Correlations with measured surface expression and normalized transport are similarly poor (not shown). Furthermore, AlphaMissense does not attempt to predict the substrate selectivity changes that impact drug transport, which means that “correct” predictions for S267X substitutions are incorrect for alternative substrates.

These pleiotropic outcomes at rheostat positions and their disparate biophysical causes may be why current computational predictors do not adequately predict substitution outcomes (e.g., AlphaMissense predictions for NTCP; Figure 9). Nor is the pleiotropy accounted for in many new sequence/function studies of proteins that use site‐saturating mutagenesis and high‐throughput biological assays (Boyle et al., 2024; Cagiada et al., 2021; Chakraborty et al., 2024; Da et al., 2021; Flynn et al., 2024; Geck et al., 2022; Mavor et al., 2016; Roscoe et al., 2013). These techniques seldom determine which functional component (e.g., binding affinity, transport rate, or allosteric regulation) is changed by a substitution or whether the levels of functional protein are altered (Swint‐Kruse & Fenton, 2024). Finally, many prediction algorithms also ignore the many occurrences of intermediate functional outcomes. These gaps in prediction and experimental measurements provide multiple routes forward for improving computational algorithms for missense mutations.

## 4 MATERIALS AND METHODS

### 4.1 Materials

Radiolabeled [^3^H]‐taurocholate (6.5 Ci/mmol) and the Optiphase HiSafe 3 Scintillation Cocktail were purchased from PerkinElmer (Boston, Massachusetts). [^3^H]‐Estrone‐3‐sulfate (50 Ci/mmol) and [^3^H]‐rosuvastatin (10 Ci/mmol) were purchased from American Radiolabeled Chemicals (Saint Louis, Missouri). Taurocholic acid sodium salt (97% pure) and estrone‐3‐sulfate sodium salt (containing 35% Tris stabilizer) were purchased from Sigma Aldrich (Saint Louis, Missouri). Rosuvastatin (98% pure) was purchased from Cayman Chemicals (Ann Arbor, Michigan). Pierce™ BCA Protein Assay Kit was purchased from Thermo Fisher Scientific (Waltham, Massachusetts). Additional analytical grade chemicals and reagents were purchased from commercial sources.

### 4.2 Bioinformatic analyses of NTPC homologs

A multiple sequence alignment (MSA) of NTCP and homologs was previously reported (Ruggiero et al., 2021). Our selection of positions 102 and 146 for the current work used this MSA and scores calculated using the original version of ConSurf (Landau et al., 2005) (http://consurf.tau.ac.il). ConSurf scores range from 1 to 9, with 1 being the least conserved position and 9 being the most conserved; these values for NTCP positions 102, 146, 267, and 271 were reported in previous publications (Ruggiero, 2021; Ruggiero et al., 2021; Ruggiero et al., 2022). After selecting positions for this work, conservation analyses were revised using the updated version of ConSurf (Yariv et al., 2023) that uses HMMER (Finn et al., 2011) for better sampling to construct sequence alignments. Revised scores are reported in Figures 1 and 7 and the new MSA is posted at https://github.com/liskinsk/NTCP-2024_MSA-data.

### 4.3 Generation of NTCP substitutions

The coding region for the His‐tagged human NTCP in the pcDNA5/FRT vector (Zhao et al., 2015) was used as a mutagenesis template. Primers were designed to flank the codons for G102 and T146 with ~20 nucleotides and ordered from Invitrogen (Carlsbad, CA) (Ruggiero et al., 2021); mutagenesis primers are listed in Table S2. For the first round of mutagenesis, the codon of interest was replaced by “NNN” to generate a library of primers for random amino acid replacement (Table S2). For a second round of mutagenesis, primers were specifically designed to generate any remaining amino acids. Mutagenesis reactions were completed using the QuikChange Lightning Multi Site‐Directed Mutagenesis Kit (Agilent Santa Clara, California) using the thermocycler parameters listed in Table S3. Sequences of the substituted NTCP coding regions were verified with Sanger sequencing (GENEWIZ, South Plainfield, NJ, USA).

### 4.4 Transient transfection of HEK293 cells

The cDNA for each substitution variant was transiently transfected into HEK293T/17 cells (American Type Culture Collection, Manassas, VA, USA) for functional or expression studies. HEK293T/17 cells were plated on poly‐d‐lysine‐coated flat bottom multi‐well cell culture plates at 35,000, 100,000, or 800,000 cells per well on 96‐, 48‐, or 6‐well plates, respectively. Twenty‐four hours later, at a cell density of 60–80 percent confluency, empty vector, wildtype NTCP, and variant NTCP plasmids were transfected into HEK293 cells using FuGENE HD and the protocol obtained from Promega. For functional studies, all plasmids were transfected in triplicate on 48‐well plates and in quadruplicate for experiments in 96‐well plates. For surface biotinylation experiments, transfections were carried out in single wells of 6‐well plates. The amount of DNA transfected per well in 96‐, 48‐, and 6‐well plates was 100 ng, 280 ng, and 3 μg, respectively. Experiments were completed 48 h post‐transfection.

### 4.5 Initial cellular uptake experiments

The cellular uptake procedure for NTCP substrates was described previously (Ruggiero et al., 2021). Briefly, cellular uptake experiments for position Y146 were completed in 48‐well plates and 96‐well plates for G102. Uptake buffer containing sodium (142 mM NaCl, 5 mM KCl, 1 mM KH_2_PO_4_, 1.2 mM MgSO_4_, 1.5 mM CaCl_2_, 5 mM glucose, and 12.5 mM HEPES, pH 7.4) was used for all washes and to prepare substrate uptake solutions. Cells were washed three times with pre‐warmed uptake buffer and then incubated for five minutes at 37°C with pre‐warmed uptake buffer containing 0.3 μCi/mL of either [^3^H]‐taurocholate, [^3^H]‐estrone‐3‐sulfate, or 0.5 μCi/mL [^3^H]‐rosuvastatin; these conditions resulted in concentrations of 30 nM, 5.8 nM, and 50 nM, respectively. Uptake was terminated by washing the cells three times with ice‐cold uptake buffer. Cells were solubilized in a 1% TX‐100 solution in PBS. An aliquot was used to determine the radioactivity in a liquid scintillation counter, and another aliquot was used to measure total protein concentrations using the Pierce BCA protein assay (Thermo Fisher Scientific). Results were calculated by first normalizing each well's measurements to the total protein and then subtracting background uptake obtained from empty vector‐transfected cells. The values for wildtype NTCP were set to 100%, and values for each substitution are reported as the percent of the wild type value.

### 4.6 Quantification of surface expression

Surface biotinylation experiments and western blotting were performed as previously described (Ruggiero et al., 2021). The following primary antibodies were used: a mouse antibody against the α subunit of Na^+^/K^+^‐ATPase (Abcam‐ab7671; 1:2000) and a mouse antibody against the His tag (Tetra·His Antibody, QIAGEN catalog no. 34670; 1:2000). An HRP‐conjugated goat anti‐mouse secondary antibody was used at 1:10,000 (Thermo Fisher Scientific, catalog no. 31430) to detect the primary antibodies. Blots were visualized using a LI‐COR Odyssey Fc (LI‐COR, Lincoln, NE), and bands were quantified using the Image Studio Lite Quantification Software (LI‐COR). The value for wildtype NTCP was set to 100% and values for each substitution are reported as the percent of the two wild‐type samples measured at the same time.

### 4.7 RheoScale calculations

The approach of determining each position's overall substitution sensitivity (i.e., a rheostat, toggle, or neutral position) allows comparison to evolutionary and structural features that can be computed from the family's multiple sequence alignment or the wild‐type structure. Prior to determining each position's overall substitution sensitivity, we first renormalized the data previously reported for substitutions at positions 267 (Ruggiero et al., 2021) and 271 (Ruggiero et al., 2022) using each day's value for the surface expression WT, so that computations are consistent with those reported here for G102X and Y146X. Updated calculations resulted in small changes in the values for S267X and N271X and provided better estimates of the propagated standard deviations for normalized transport. All other results and conclusions of the prior publications remain the same. Updated values for S267X and N271X are reported in Table S1 along with the values for G102X and Y146X.

To quantify and compare overall substitution outcomes, we utilized the RheoScale calculator (Hodges et al., 2018) for each of the four experimental outcomes at each position. This calculator uses a histogram‐based analysis to calculate rheostat scores for each position. In this analysis, one histogram bin includes the wildtype value. Substitutions that fall into this bin are functionally similar to wildtype (i.e., neutral); if ≥70% of a position's set of substitutions are neutral substitutions, the position itself is classified as neutral (Martin et al., 2020). Another bin includes the non‐functional substitutions (or those with no detectable protein expression); if at least two‐thirds of the substitutions fall into this bin, the position is considered a toggle position (Wu et al., 2019). The remaining bins are filled with substitutions that have intermediate loss or enhanced outcomes. The total number of bins sampled by a set of substitutions is used to calculate a “rheostat” score; a perfect rheostat position (substitutions sample all possible outcomes) has a score of 1. If the substitutions for a position sample at least half of the accessible range, the position is considered a rheostat position (Hodges et al., 2018). In control calculations for NTCP analyses, the histogram bin number was varied between 5 and 11, which showed that it did not affect position class assignments. Values reported in Figure 7 were computed with 10 bins.

### 4.8 Statistical analyses

Statistical analyses and correlation comparisons were performed using GraphPad Prism 8 (uncorrected uptake) or 10.1.2 (surface expression) (GraphPad Software Inc., San Diego, CA). Significance was determined using one‐way ANOVA followed by Dunnett's post hoc test for multiple comparisons. Results were considered significantly different at *p* < 0.05.

## AUTHOR CONTRIBUTIONS

**Liskin Swint‐Kruse:** Conceptualization; funding acquisition; writing – original draft; supervision; methodology; formal analysis; visualization; resources; data curation; validation; investigation. **Melissa J. Ruggiero:** Investigation; conceptualization; writing – review and editing; formal analysis; visualization. **Bruno Hagenbuch:** Conceptualization; funding acquisition; writing – review and editing; validation; formal analysis; resources; supervision; data curation; project administration; visualization.

## Supporting information


**Data S1.** Supporting information.

**Table S1.** Supporting information.

## Data Availability

The data that support the findings of this study are openly available in NTCP‐2025‐MSA and data at https://github.com/liskinsk/NTCP-2024_MSA-data.

## References

[pro70313-bib-0001] An P , Zeng Z , Winkler CA . The loss‐of‐function S267F variant in HBV receptor NTCP reduces human risk for HBV infection and disease progression. J Infect Dis. 2018;218:1404–1410. 29905807 10.1093/infdis/jiy355 PMC6151084

[pro70313-bib-0002] Anwer MS , Stieger B . Sodium‐dependent bile salt transporters of the SLC10A transporter family: more than solute transporters. Pflugers Arch. 2014;466:77–89. 24196564 10.1007/s00424-013-1367-0 PMC3877701

[pro70313-bib-0003] Appelman MD , Chakraborty A , Protzer U , McKeating JA , van de Graaf SF . N‐glycosylation of the Na^+^‐taurocholate cotransporting polypeptide (NTCP) determines its trafficking and stability and is required for hepatitis B virus infection. PLoS One. 2017;12:e0170419. 28125599 10.1371/journal.pone.0170419 PMC5268470

[pro70313-bib-0004] Ashkenazy H , Abadi S , Martz E , Chay O , Mayrose I , Pupko T , et al. ConSurf 2016: an improved methodology to estimate and visualize evolutionary conservation in macromolecules. Nucleic Acids Res. 2016;44:W344–W350. 27166375 10.1093/nar/gkw408 PMC4987940

[pro70313-bib-0005] Bantis LE , Parente DJ , Fenton AW , Swint‐Kruse L . “Multiplex” rheostat positions cluster around allosterically critical regions of the lactose repressor protein. bioRxiv. 2020; 10.1101/2020.11.17.386979.

[pro70313-bib-0006] Bendell CJ , Liu S , Aumentado‐Armstrong T , Istrate B , Cernek PT , Khan S , et al. Transient protein‐protein interface prediction: datasets, features, algorithms, and the RAD‐T predictor. BMC Bioinformatics. 2014;15:82. 24661439 10.1186/1471-2105-15-82 PMC4021185

[pro70313-bib-0007] Boyle GE , Sitko K , Galloway JG , Haddox HK , Bianchi AH , Dixon A , et al. Deep mutational scanning of CYP2C19 in human cells reveals a substrate specificity‐abundance tradeoff. Genetics. 2024;228:iyae156. 39319420 10.1093/genetics/iyae156 PMC11538415

[pro70313-bib-0008] Cagiada M , Johansson KE , Valanciute A , Nielsen SV , Hartmann‐Petersen R , Yang JJ , et al. Understanding the origins of loss of protein function by analyzing the effects of thousands of variants on activity and abundance. Mol Biol Evol. 2021;38:3235–3246. 33779753 10.1093/molbev/msab095 PMC8321532

[pro70313-bib-0009] Campitelli P , Ross D , Swint‐Kruse L , Ozkan SB . Dynamics‐based protein network features accurately discriminate neutral and rheostat positions. Biophys J. 2024;123:3612–3626. 39277794 10.1016/j.bpj.2024.09.013 PMC11494493

[pro70313-bib-0010] Campitelli P , Swint‐Kruse L , Ozkan S . Substitutions at non‐conserved rheostat positions modulate function by re‐wiring long‐range, dynamic interactions. Mol Biol Evol. 2021;38:201–214. 32780837 10.1093/molbev/msaa202 PMC7783170

[pro70313-bib-0011] Chakraborty S , Ahler E , Simon JJ , Fang L , Potter ZE , Sitko KA , et al. Profiling of drug resistance in Src kinase at scale uncovers a regulatory network coupling autoinhibition and catalytic domain dynamics. Cell Chem Biol. 2024;31:207–220. 37683649 10.1016/j.chembiol.2023.08.005 PMC10902203

[pro70313-bib-0012] Cheng J , Novati G , Pan J , Bycroft C , Žemgulytė A , Applebaum T , et al. Accurate proteome‐wide missense variant effect prediction with AlphaMissense. Science. 2023;381:eadg7492. 37733863 10.1126/science.adg7492

[pro70313-bib-0013] Chi PB , Liberles DA . Selection on protein structure, interaction, and sequence. Protein Sci. 2016;25:1168–1178. 26808055 10.1002/pro.2886 PMC4918422

[pro70313-bib-0014] Clarke JD , Novak P , Lake AD , Hardwick RN , Cherrington NJ . Impaired N‐linked glycosylation of uptake and efflux transporters in human non‐alcoholic fatty liver disease. Liver Int. 2017;37:1074–1081. 28097795 10.1111/liv.13362 PMC5479731

[pro70313-bib-0015] Claro da Silva T , Polli JE , Swaan PW . The solute carrier family 10 (SLC10): beyond bile acid transport. Mol Aspects Med. 2013;34:252–269. 23506869 10.1016/j.mam.2012.07.004 PMC3602841

[pro70313-bib-0016] Kuang D , Weile J , Kishore N , Nguyen M , Rubin AF , Fields S , et al. MaveRegistry: a collaboration platform for multiplexed assays of variant effect. Bioinformatics. 2021;37:3382–3383. 33774657 10.1093/bioinformatics/btab215 PMC8504617

[pro70313-bib-0017] Doring B , Lutteke T , Geyer J , Petzinger E . The SLC10 carrier family: transport functions and molecular structure. Curr Top Membr. 2012;70:105–168. 23177985 10.1016/B978-0-12-394316-3.00004-1

[pro70313-bib-0018] Fenton KD , Meneely KM , Wu T , Martin TA , Swint‐Kruse L , Fenton AW , et al. Substitutions at a rheostat position in human aldolase a cause a shift in the conformational population. Protein Sci. 2022;31:357–370. 34734672 10.1002/pro.4222 PMC8819835

[pro70313-bib-0019] Fenton AW , Page BM , Spellman‐Kruse A , Hagenbuch B , Swint‐Kruse L . Rheostat positions: a new classification of protein positions relevant to pharmacogenomics. Med Chem Res. 2020;29:1133–1146. 32641900 10.1007/s00044-020-02582-9 PMC7276102

[pro70313-bib-0020] Finn RD , Clements J , Eddy SR . HMMER web server: interactive sequence similarity searching. Nucleic Acids Res. 2011;39:W29–W37. 21593126 10.1093/nar/gkr367 PMC3125773

[pro70313-bib-0021] Flynn JM , Zvornicanin SN , Tsepal T , Shaqra AM , Kurt Yilmaz N , Jia W , et al. Contributions of hyperactive mutations in M(pro) from SARS‐CoV‐2 to drug resistance. ACS Infect Dis. 2024;10:1174–1184. 38472113 10.1021/acsinfecdis.3c00560 PMC11179160

[pro70313-bib-0022] Geck RC , Boyle G , Amorosi CJ , Fowler DM , Dunham MJ . Measuring pharmacogene variant function at scale using multiplexed assays. Annu Rev Pharmacol Toxicol. 2022;62:531–550. 34516287 10.1146/annurev-pharmtox-032221-085807

[pro70313-bib-0023] Hagenbuch B , Meier PJ . Molecular cloning, chromosomal localization, and functional characterization of a human liver Na^+^/bile acid cotransporter. J Clin Invest. 1994;93:1326–1331. 8132774 10.1172/JCI117091 PMC294097

[pro70313-bib-0024] Ho RH , Leake BF , Roberts RL , Lee W , Kim RB . Ethnicity‐dependent polymorphism in Na^+^‐taurocholate cotransporting polypeptide (SLC10A1) reveals a domain critical for bile acid substrate recognition. J Biol Chem. 2004;279:7213–7222. 14660639 10.1074/jbc.M305782200

[pro70313-bib-0025] Ho RH , Tirona RG , Leake BF , Glaeser H , Lee W , Lemke CJ , et al. Drug and bile acid transporters in rosuvastatin hepatic uptake: function, expression, and pharmacogenetics. Gastroenterology. 2006;130:1793–1806. 16697742 10.1053/j.gastro.2006.02.034

[pro70313-bib-0026] Hodges AM , Fenton AW , Dougherty LL , Overholt AC , Swint‐Kruse L . RheoScale: a tool to aggregate and quantify experimentally determined substitution outcomes for multiple variants at individual protein positions. Hum Mutat. 2018;39:1814–1826. 30117637 10.1002/humu.23616 PMC6602090

[pro70313-bib-0027] Hou Q , Rooman M , Pucci F . Enzyme stability‐activity trade‐off: new insights from protein stability weaknesses and evolutionary conservation. J Chem Theory Comput. 2023;19:3664–3671. 37276063 10.1021/acs.jctc.3c00036

[pro70313-bib-0028] Kariyawasam NL , Sivchenko A , Swint‐Kruse L , Smith PE . Substitutions at rheostat position 52 of LacI have long‐range effects on the LacI conformational landscape. Biophys Chem. 2025;320‐321:107414. 10.1016/j.bpc.2025.107414 PMC11893255 39987706

[pro70313-bib-0029] Khersonsky O , Rothlisberger D , Dym O , Albeck S , Jackson CJ , Baker D , et al. Evolutionary optimization of computationally designed enzymes: Kemp eliminases of the KE07 series. J Mol Biol. 2010;396:1025–1042. 20036254 10.1016/j.jmb.2009.12.031

[pro70313-bib-0030] Klesmith JR , Bacik JP , Wrenbeck EE , Michalczyk R , Whitehead TA . Trade‐offs between enzyme fitness and solubility illuminated by deep mutational scanning. Proc Natl Acad Sci U S A. 2017;114:2265–2270. 28196882 10.1073/pnas.1614437114 PMC5338495

[pro70313-bib-0031] Kullak‐Ublick GA , Glasa J , Boker C , Oswald M , Grutzner U , Hagenbuch B , et al. Chlorambucil‐taurocholate is transported by bile acid carriers expressed in human hepatocellular carcinomas. Gastroenterology. 1997;113:1295–1305. 9322525 10.1053/gast.1997.v113.pm9322525

[pro70313-bib-0032] Kumar S , Bansal M . Dissecting alpha‐helices: position‐specific analysis of alpha‐helices in globular proteins. Proteins. 1998;31:460–476. 9626705 10.1002/(sici)1097-0134(19980601)31:4<460::aid-prot12>3.0.co;2-d

[pro70313-bib-0033] Kunz S , Soppa l , Leidolf R , Neubauer A , Lütteke T , Glebe D , et al. Interaction between W41 of the hepatitis B virus preS1 surface peptide and Y146/F274 of the cellular receptor molecule NTCP is essential for virus entry. Mol Pharmacol. 2025;107:100069.40925208 10.1016/j.molpha.2025.100069

[pro70313-bib-0034] Landau M , Mayrose I , Rosenberg Y , Glaser F , Martz E , Pupko T , et al. ConSurf 2005: the projection of evolutionary conservation scores of residues on protein structures. Nucleic Acids Res. 2005;33:W299–W302. 15980475 10.1093/nar/gki370 PMC1160131

[pro70313-bib-0035] Lee HW , Park HJ , Jin B , Dezhbord M , Kim DY , Han KH , et al. Effect of S267F variant of NTCP on the patients with chronic hepatitis B. Sci Rep. 2017;7:17634. 29247233 10.1038/s41598-017-17959-x PMC5732244

[pro70313-bib-0036] Liu H , Irobalieva RN , Bang‐Sorensen R , Nosol K , Mukherjee S , Agrawal P , et al. Structure of human NTCP reveals the basis of recognition and sodium‐driven transport of bile salts into the liver. Cell Res. 2022;32:773–776. 35726088 10.1038/s41422-022-00680-4 PMC9343345

[pro70313-bib-0037] Martin TA , Wu T , Tang Q , Dougherty LL , Parente DJ , Swint‐Kruse L , et al. Identification of biochemically neutral positions in liver pyruvate kinase. Proteins. 2020;88:1340–1350. 32449829 10.1002/prot.25953 PMC8990530

[pro70313-bib-0038] Mavor D , Barlow K , Thompson S , Barad BA , Bonny AR , Cario CL , et al. Determination of ubiquitin fitness landscapes under different chemical stresses in a classroom setting. Elife. 2016;5:e15802. 27111525 10.7554/eLife.15802 PMC4862753

[pro70313-bib-0039] Meinhardt S , Manley MW Jr , Parente DJ , Swint‐Kruse L . Rheostats and toggle switches for modulating protein function. PLoS One. 2013;8:e83502. 24386217 10.1371/journal.pone.0083502 PMC3875437

[pro70313-bib-0040] Miller M , Bromberg Y , Swint‐Kruse L . Computational predictors fail to identify amino acid substitution effects at rheostat positions. Sci Rep. 2017;7:41329. 28134345 10.1038/srep41329 PMC5278360

[pro70313-bib-0041] Page BM , Martin TA , Wright CL , Fenton LA , Villar MT , Tang Q , et al. Odd one out? Functional tuning of *Zymomonas mobilis* pyruvate kinase is narrower than its allosteric, human counterpart. Protein Sci. 2022;31:e4336. 35762709 10.1002/pro.4336 PMC9202079

[pro70313-bib-0042] Pan W , Song IS , Shin HJ , Kim MH , Choi YL , Lim SJ , et al. Genetic polymorphisms in Na^+^‐taurocholate co‐transporting polypeptide (NTCP) and ileal apical sodium‐dependent bile acid transporter (ASBT) and ethnic comparisons of functional variants of NTCP among Asian populations. Xenobiotica. 2011;41:501–510. 21341987 10.3109/00498254.2011.555567

[pro70313-bib-0043] Park JH , Iwamoto M , Yun JH , Uchikubo‐Kamo T , Son D , Jin Z , et al. Structural insights into the HBV receptor and bile acid transporter NTCP. Nature. 2022;606:1027–1031. 35580630 10.1038/s41586-022-04857-0 PMC9242859

[pro70313-bib-0044] Pettersen EF , Goddard TD , Huang CC , Couch GS , Greenblatt DM , Meng EC , et al. UCSF chimera – a visualization system for exploratory research and analysis. J Comput Chem. 2004;25:1605–1612. 15264254 10.1002/jcc.20084

[pro70313-bib-0045] Roscoe BP , Thayer KM , Zeldovich KB , Fushman D , Bolon DNA . Analyses of the effects of all ubiquitin point mutants on yeast growth rate. J Mol Biol. 2013;425:1363–1377. 23376099 10.1016/j.jmb.2013.01.032 PMC3615125

[pro70313-bib-0046] Ruggiero MJ . Characterization of the function and expression of variants at potential rheostat, toggle, and neutral positions in the Na^+^/taurocholate cotransporting polypeptide (NTCP). Ph.D., University of Kansas. 2021.

[pro70313-bib-0047] Ruggiero MJ , Malhotra S , Fenton AW , Swint‐Kruse L , Karanicolas J , Hagenbuch B . A clinically relevant polymorphism in the Na(+)/taurocholate cotransporting polypeptide (NTCP) occurs at a rheostat position. J Biol Chem. 2021;296:100047. 33168628 10.1074/jbc.RA120.014889 PMC7948949

[pro70313-bib-0048] Ruggiero MJ , Malhotra S , Fenton AW , Swint‐Kruse L , Karanicolas J , Hagenbuch B . Structural plasticity is a feature of rheostat positions in the human Na(+)/taurocholate cotransporting polypeptide (NTCP). Int J Mol Sci. 2022;23:3211. 35328632 10.3390/ijms23063211 PMC8954283

[pro70313-bib-0049] Shionoya K , Park JH , Ekimoto T , Takeuchi JS , Mifune J , Morita T , et al. Structural basis for hepatitis B virus restriction by a viral receptor homologue. Nat Commun. 2024;15:9241. 39455604 10.1038/s41467-024-53533-6 PMC11511851

[pro70313-bib-0050] Shneider BL , Fox VL , Schwarz KB , Watson CL , Ananthanarayanan M , Thevananther S , et al. Hepatic basolateral sodium‐dependent‐bile acid transporter expression in two unusual cases of hypercholanemia and in extrahepatic biliary atresia. Hepatology. 1997;25:1176–1183. 9141436 10.1002/hep.510250521

[pro70313-bib-0051] Slijepcevic D , Kaufman C , Wichers CG , Gilglioni EH , Lempp FA , Duijst S , et al. Impaired uptake of conjugated bile acids and hepatitis b virus pres1‐binding in Na(+)‐taurocholate cotransporting polypeptide knockout mice. Hepatology. 2015;62:207–219. 25641256 10.1002/hep.27694 PMC4657468

[pro70313-bib-0052] Sreenivasan S , Fontes JD , Swint‐Kruse L . Dissecting the effects of single amino acid substitutions in SARS‐CoV2 Mpro. Protein Sci 2025; 34:e70225. 40671356 10.1002/pro.70225 PMC12267653

[pro70313-bib-0053] Sreenivasan S , Heffren P , Suh KS , Rodnin MV , Kosa E , Fenton AW , et al. The intrinsically disordered transcriptional activation domain of CIITA is functionally tuneable by single substitutions: an exception or a new paradigm? Protein Sci. 2024;33:e4863. 38073129 10.1002/pro.4863 PMC10806935

[pro70313-bib-0054] Swint‐Kruse L , Fenton AW . Rheostats, toggles, and neutrals, oh my! A new framework for understanding how amino acid changes modulate protein function. J Biol Chem. 2024;300:105736. 38336297 10.1016/j.jbc.2024.105736 PMC10914490

[pro70313-bib-0055] Swint‐Kruse L , Martin TA , Page BM , Wu T , Gerhart PM , Dougherty LL , et al. Rheostat functional outcomes occur when substitutions are introduced at nonconserved positions that diverge with speciation. Protein Sci. 2021;30:1833–1853. 34076313 10.1002/pro.4136 PMC8376419

[pro70313-bib-0056] Tungtur S , Schwingen KM , Riepe JJ , Weeramange CJ , Swint‐Kruse L . Homolog comparisons further reconcile in vitro and in vivo correlations of protein activities by revealing over‐looked physiological factors. Protein Sci. 2019;28:1806–1818. 31351028 10.1002/pro.3695 PMC6739814

[pro70313-bib-0057] Wimley WC , Creamer TP , White SH . Solvation energies of amino acid side chains and backbone in a family of host‐guest pentapeptides. Biochemistry. 1996;35:5109–5124. 8611495 10.1021/bi9600153

[pro70313-bib-0058] Wu T , Swint‐Kruse L , Fenton AW . Functional tunability from a distance: rheostat positions influence allosteric coupling between two distant binding sites. Sci Rep. 2019;9:16957. 31740686 10.1038/s41598-019-53464-z PMC6861286

[pro70313-bib-0059] Xie WJ , Asadi M , Warshel A . Enhancing computational enzyme design by a maximum entropy strategy. Proc Natl Acad Sci USA. 2022;119:e2122355119. 35135886 10.1073/pnas.2122355119 PMC8851541

[pro70313-bib-0060] Xie WJ , Warshel A . Natural evolution provides strong hints about laboratory evolution of designer enzymes. Proc Natl Acad Sci USA. 2022;119:e2207904119. 35901204 10.1073/pnas.2207904119 PMC9351539

[pro70313-bib-0061] Yariv B , Yariv E , Kessel A , Masrati G , Chorin AB , Martz E , et al. Using evolutionary data to make sense of macromolecules with a “face‐lifted” ConSurf. Protein Sci. 2023;32:e4582. 36718848 10.1002/pro.4582 PMC9942591

[pro70313-bib-0062] Zhao W , Zitzow JD , Ehresman DJ , Chang SC , Butenhoff JL , Forster J , et al. Na^+^/taurocholate cotransporting polypeptide and apical sodium‐dependent bile acid transporter are involved in the disposition of perfluoroalkyl sulfonates in humans and rats. Toxicol Sci. 2015;146:363–373. 26001962 10.1093/toxsci/kfv102 PMC4607751

