# Supplementary material for: Amino acid substitutions at rheostat positions in the Na+/taurocholate cotransporting polypeptide substrate channel have pleiotropic effects
Source: Protein Sci. 2025 Oct 14;34(11):e70313. doi: 10.1002/pro.70313 (PMC12521614; doi:10.1002/pro.70313)
Supplement: Supplementary file 1 — Data S1. Supporting information. [file PRO-34-e70313-s001.docx]

Supplement to: Amino acid substitutions at rheostat positions in the NTCP substrate channel have pleiotropic effects

Liskin Swint-Kruse, Melissa J Ruggiero, Bruno Hagenbuch

**Supplemental Figure 1.** The NTCP model used to predict structural plasticity to substitutions and a high prevalence of rheostat positions (Ruggiero et al. 2022) is in good agreement with the cryo-EM structure (PDB 7zyi). The model of the inward-open structure is shown with a black ribbon and black ball-and-stick for the four positions in this and our prior studies. The cryo-EM structure is shown with a gray ribbon and space filling for these same four positions. Of these, three have overlapping side chains. The exoplasmic loop containing Y146 is in a different location (green). Differences are also noted in the helices that form the “panel” on the lower right of the structure. The other 13 positions predicted to have plasticity by computation are not shown. This figure was rendered with UCSF chimera (Pettersen et al. 2004).


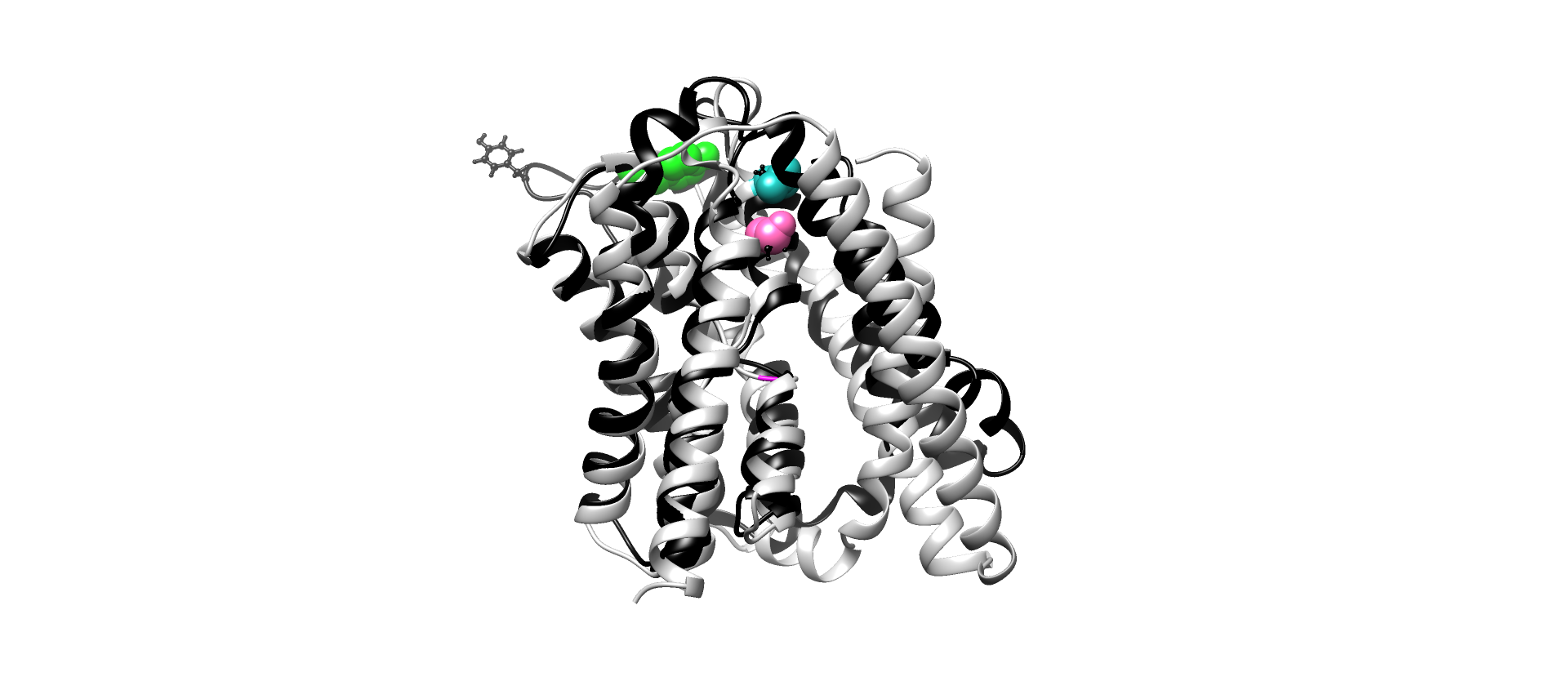


**Supplemental Table 1**. All data for the positions reported and discussed in this study are contained in an Excel™ spreadsheet that is associated with this study and posted at https://github.com/liskinsk/NTCP-2024_MSA-data.

**Supplemental Figure 2**. Representative western blots of total protein expression of wildtype and G102 variants in transiently transfected HEK293 cells. Total protein samples from surface biotinylation experiments were electrophoresed on 4-20% gradient polyacrylamide gels. Separated protein was then transferred to nitrocellulose membranes and then probed with an anti-Na^+^/K^+^-ATPase antibody as a loading control and the Tetra-His antibody to detect His-tagged NTCP. The different bands seen for wild type and the substitutions correspond most likely to different glycosylation states and/or degradation products of NTCP.


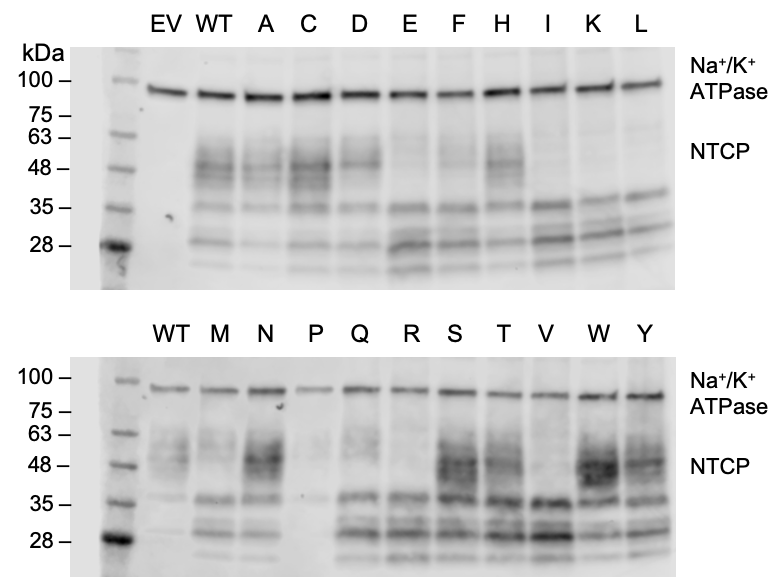


**Supplemental Figure 3.** Representative western blots of total protein expression of wildtype and Y146 variants in transiently transfected HEK293 cells. Total protein samples from surface biotinylation experiments were electrophoresed on 4-20% gradient polyacrylamide gels. Separated protein was then transferred to nitrocellulose membranes and then probed with an anti-Na^+^/K^+^-ATPase antibody as a loading control and a Tetra-His antibody to detect His-tagged NTCP. The different bands seen for wild type and the substitutions correspond most likely to different glycosylation states and/or degradation products of NTCP.


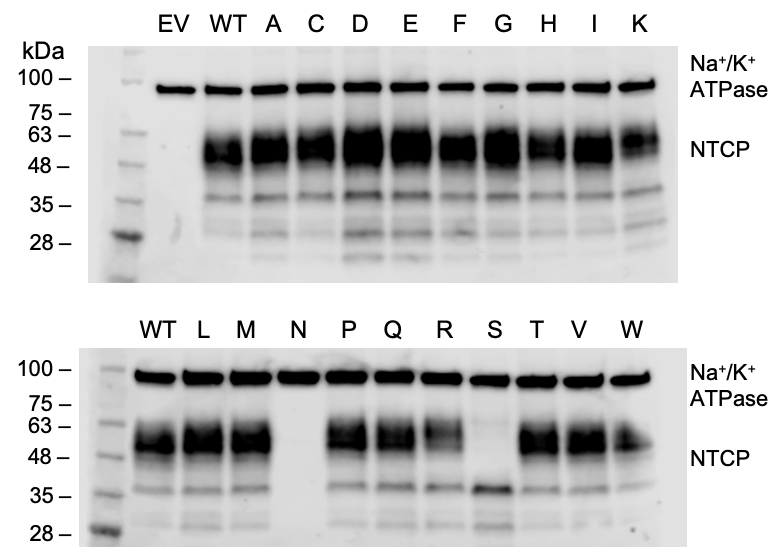


**Supplemental Figure 4**. G102X Surface expression versus corrected uptake. The lack of correlation between these parameters suggests that the two properties are controlled by independent biophysical parameters. Plots are shown here on log scale; correlations were performed in Graphpad Prism on the unstransformed data.

**
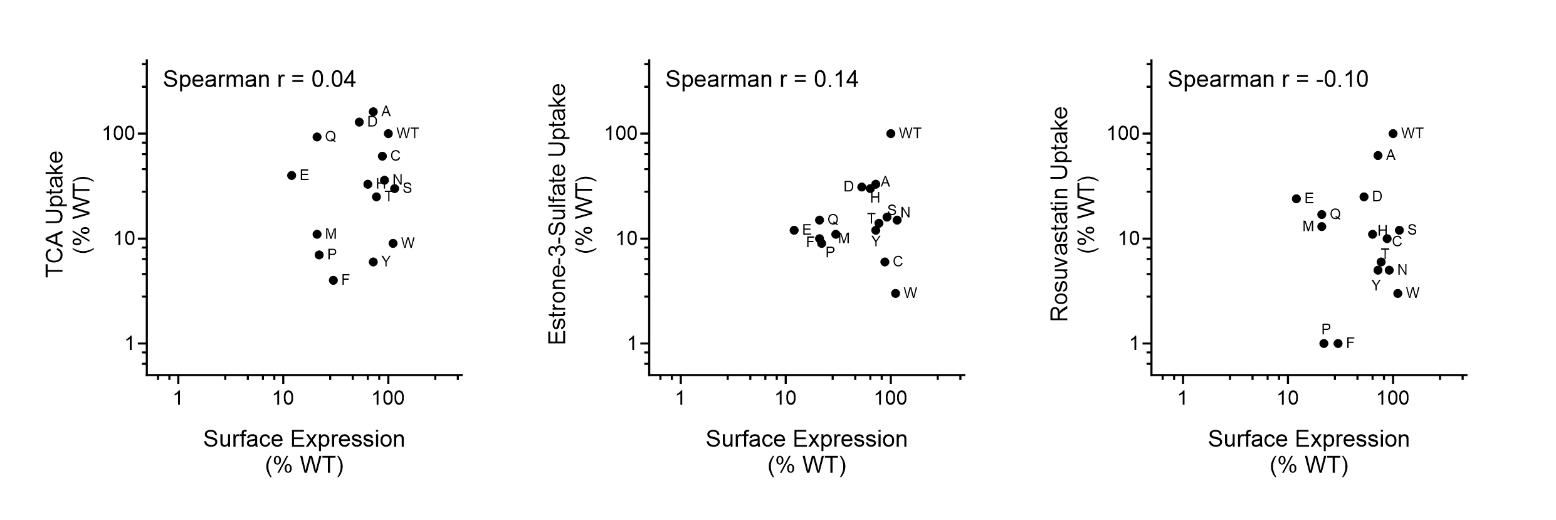
**

**Supplemental Figure 5.** Correlation of G102X outcomes with amino acid properties. Since substitutions showed comparable effects on the three substrates (main text Figure 5), structural analyses are shown for TCA, which had the greatest (and thus most reliably measured) uptake. Of the features discussed in the main text, only those shown below have correlation coefficients > 0.5. Spearman correlation coefficients are used because the relationship is not necessarily expected to be linear. Units for solvent-exposed surface area (amino acid size) are in Å^2^, as measured in the context of a Gly-X-Gly peptide (Bendell et al. 2014). The citation for the hydrophobicity scale is (Wimley et al. 1996) and for the helical propensity is (Kumar and Bansal 1998). Plots are shown here on log scale; correlations were performed in Graphpad Prism on the unstransformed data.


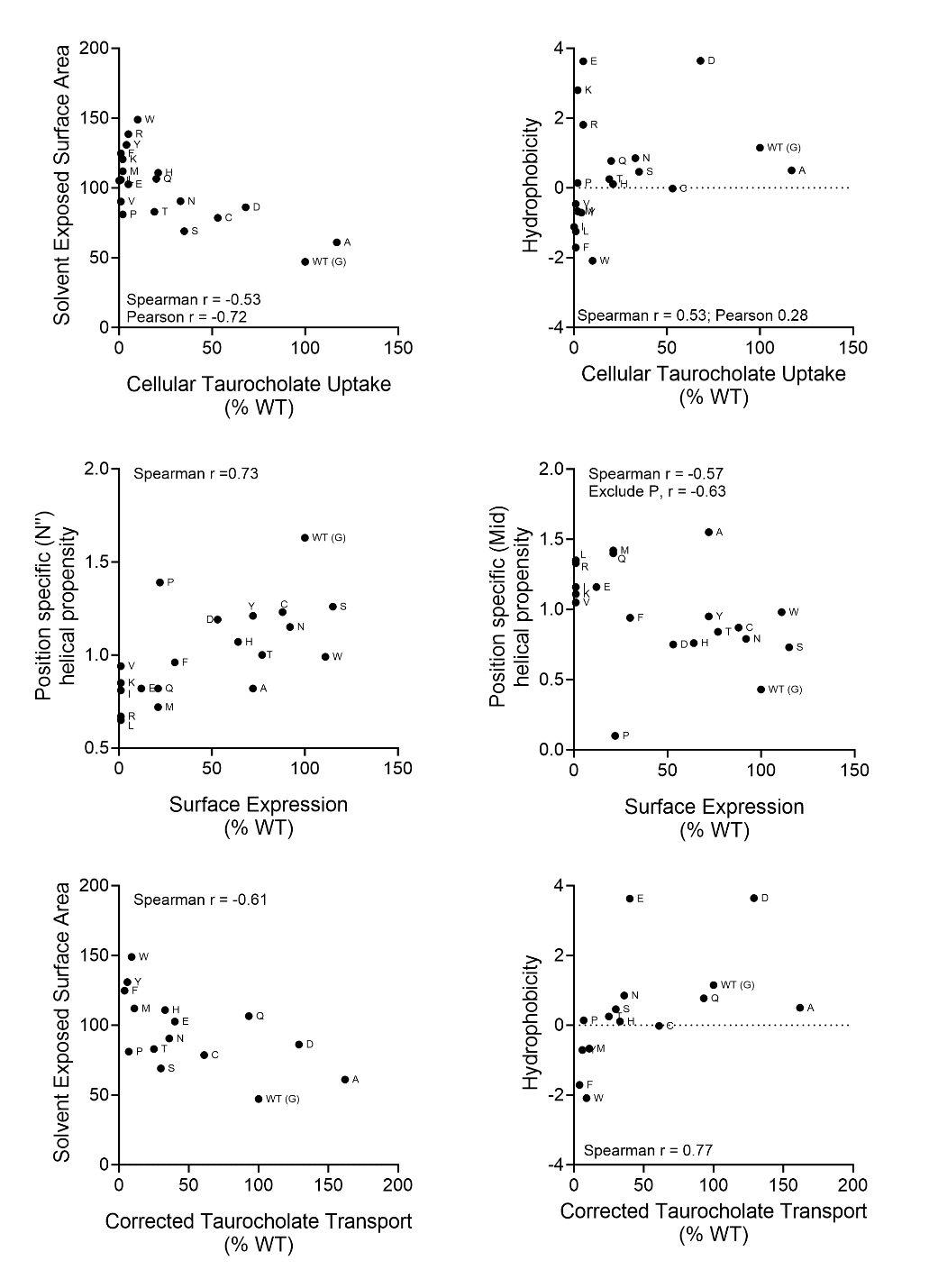


**Supplemental Figure 6.**  Correlation of N271X outcomes with amino acid properties. Since substitutions showed comparable effects on the three substrates (main text Figure 5), structural analyses are shown for TCA, which had the greatest (and thus most reliably measured) uptake. Of the features discussed in the main text, only those shown below have correlation coefficients > 0.5. Spearman correlation coefficients are used because the relationship is not necessarily expected to be linear. Plots are shown here on log scale; correlations were performed in Graphpad Prism on the unstransformed data.


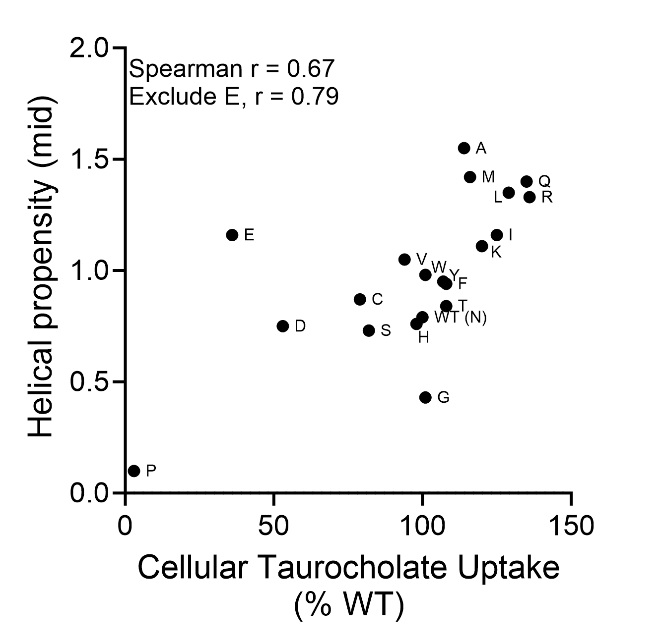


**Supplemental Table 2.** Primers used for site-directed mutagenesis at positions G102 and Y145

| G102X | 5’-TGTGGCTGCTCACCTGGA**NNN**AACCTGTCCAATGTCTTCAGTCTG-3’ |
| --- | --- |
| G102A | 5’-TGTGGCTGCTCACCTGGA**GCC**AACCTGTCCAATGTCTTCAGTCTG-3’ |
| G102R | 5’-TGTGGCTGCTCACCTGGA**AGA**AACCTGTCCAATGTCTTCAGTCTG-3’ |
| G102C | 5’-TGTGGCTGCTCACCTGGA**TGC**AACCTGTCCAATGTCTTCAGTCTG-3’ |
| G102E | 5’-TGTGGCTGCTCACCTGGA**GAG**AACCTGTCCAATGTCTTCAGTCTG-3’ |
| G102Q | 5’-TGTGGCTGCTCACCTGGA**CAG**AACCTGTCCAATGTCTTCAGTCTG-3’ |
| G102H | 5’-TGTGGCTGCTCACCTGGA**CAC**AACCTGTCCAATGTCTTCAGTCTG-3’ |
| G102K | 5’-TGTGGCTGCTCACCTGGA**AAG**AACCTGTCCAATGTCTTCAGTCTG-3’ |
| G102M | 5’-TGTGGCTGCTCACCTGGA**ATG**AACCTGTCCAATGTCTTCAGTCTG-3’ |
| Y146X | 5’-TACATCTACTCCAGGGGGATC**NNN**GATGGGGACCTGAAGGAC-3’ |
| Y146A | 5’-TACATCTACTCCAGGGGGATC**GCC**GATGGGGACCTGAAGGAC-3’ |
| Y146Q | 5’-TACATCTACTCCAGGGGGATC**CAG**GATGGGGACCTGAAGGAC-3’ |
| Y146H | 5’-TACATCTACTCCAGGGGGATC**CAC**GATGGGGACCTGAAGGAC-3’ |
| Y146L | 5’-TACATCTACTCCAGGGGGATC**CTG**GATGGGGACCTGAAGGAC-3’ |
| Y146M | 5’-TACATCTACTCCAGGGGGATC**ATG**GATGGGGACCTGAAGGAC-3’ |
| Y146F | 5’-TACATCTACTCCAGGGGGATC**TTC**GATGGGGACCTGAAGGAC-3’ |
| Y146P | 5’-TACATCTACTCCAGGGGGATC**CCC**GATGGGGACCTGAAGGAC-3’ |

**Supplemental Table 3**. Mutagenesis Thermocycling Parameters

| # of Cycles | Temp in °C | Length of Cycle (Time) |
| --- | --- | --- |
| 1 | 95 | 2 minutes |
| 30 | 95 | 20 seconds |
|  | 55 | 30 seconds |
|  | 65 | 2 minutes 50 seconds (30sec/kb of plasmid - NTCP ~ 5.6 kb) |
| 1 | 65 | 5 minutes |
| 1 | 4 | ∞ hold |

**References cited**

1. Ruggiero, M. J., S. Malhotra, A. W. Fenton, L. Swint-Kruse, J. Karanicolas and B. Hagenbuch (2022). Structural Plasticity Is a Feature of Rheostat Positions in the Human Na^+^/Taurocholate Cotransporting Polypeptide (NTCP). Int J Mol Sci 23.
2. Pettersen, E. F., T. D. Goddard, C. C. Huang, G. S. Couch, D. M. Greenblatt, E. C. Meng and T. E. Ferrin (2004). UCSF Chimera--a visualization system for exploratory research and analysis. J Comput Chem 25:1605-1612.
3. Bendell, C. J., S. Liu, T. Aumentado-Armstrong, B. Istrate, P. T. Cernek, S. Khan, S. Picioreanu, M. Zhao and R. A. Murgita (2014). Transient protein-protein interface prediction: datasets, features, algorithms, and the RAD-T predictor. BMC Bioinformatics 15:82.
4. Wimley, W. C., T. P. Creamer and S. H. White (1996). Solvation energies of amino acid side chains and backbone in a family of host-guest pentapeptides. Biochemistry 35:5109-5124.
5. Kumar, S. and M. Bansal (1998). Dissecting alpha-helices: position-specific analysis of alpha-helices in globular proteins. Proteins 31:460-476.
